# State-of-the-art requirements verification process applied to numerical finite-element modelling of the deep excavation in soft soils

**DOI:** 10.1371/journal.pone.0298061

**Published:** 2024-04-04

**Authors:** Zoa Ambassa, Jean Chills Amba

**Affiliations:** 1 Laboratory of Energy Modeling Materials and Methods (E3M), National Higher Polytechnic School of Douala, University of Douala, Douala, Cameroon; 2 Department of Civil Engineering, National Higher Polytechnic School of Douala, University of Douala, Douala, Cameroon; China University of Mining and Technology, CHINA

## Abstract

The study presents state-of-the-art requirements verification process for the prediction of the stability of the multi-staged deep excavation in submerged soft soil retained by stell sheet pile walls structures applied at the development of elasto-plastic finite element calculation method performed from Cast3M and Plaxis FE codes. Optimization numerical calculation results are proposed for retained walls design and construction on the basis of the horizontal displacement, earth and water pressures measurements. The transformation of the geometry and stiffness of the stell sheet pile walls to the retaining walls of an equivalent bending stiffness on the one hand and regular geometric shapes allowed in this paper to overcome the difficulties of modelling these stell sheet pile walls in 2D with irregular shapes. The horizontal deflection of the wall, the vertical displacement behind the walls, and the settlement of the excavation bottom are given. They have been compared by those obtained by various authors around the world. The results of this approach are satisfactory in view of the horizontal displacement curves obtained on the stell sheet pile walls compared by the measures.

## 1. Introduction and literature review

### 1.1 Introduction

For decades, the majority of retained walls stability analyses performed in practice still use traditional limit equilibrium approaches that have remained essentially unchanged. The Finite Element Method represents a powerful alternative approach for retained wall analysis which is accurate, versatile and requires fewer a priori assumptions, especially, regarding the failure mechanism. Elasto-plastic analysis of geotechnical problems using the Finite Element (FE) Method has been widely accepted in the research arena for many years; however, its routine use in geotechnical practice for retaining stability analysis still remains limited. The reason for this lack of acceptance is not entirely clear; however, advocates of FE techniques in academe must take some responsibility. Practising engineers are often sceptical of the need for such complexity, especially in view of the poor quality of soil property data often available from routine site investigations [1–3]. Although this scepticism is often warranted, there are certain types of geotechnical problem for which the FE approach offers real benefits. The challenge for an experienced engineer is to know which kind of problem would benefit from a FE treatment and which would not. In general, linear problems such as the prediction of settlements and strains, the calculation of flow quantities due to steady seepage or the study of transient effects due to consolidation are all highly amenable to solution by Finite Elements. Traditional approaches involving charts, tables or graphical methods will often be adequate for routine problems but the Finite Elements approach may be valuable if awkward geometries or material variations are encountered which are not covered by traditional chart solutions.

The use of nonlinear analysis in routine geotechnical practice is harder to justify, because there is usually a significant increase in complexity which is more likely to require the help of a modelling specialist [4]. Nonlinear analyses are inherently iterative in nature, because the material properties and/ or the geometry of the problem are themselves a function of the ‘solution’. Objections to nonlinear analyses on the grounds that they require excessive computational power, however, have been largely overtaken by developments in, and falling costs of, computer hardware. The submerged soft soil retained by stell sheet pile walls represents an area of geotechnical analysis in which a nonlinear FE approach offers real benefits over existing methods. As this paper will show, retained wall analysis under multi-staged deep excavation on soft soils by elasto-plastic Finite Elements is accurate, robust and simple enough for routine use by practising engineers.

The objective of this work is the development of an elasto-plastic calculation method for the prediction of the stability of the multi-staged deep excavation in submerged multi-layered soft soil retained by stell sheet pile walls structures by Finite Element technique thanks to with methodological and sophisticated computing from Cast3M FE code. Optimization numerical calculation results are proposed for retained stell sheet pile walls design and construction on the basis of the horizontal displacements measurements. The paper describes several standards in geotechnical practice of Finite Element retained wall analysis with comparison against field test measurements. In this context this paper improve the state-of-the art-requirements verification process. We approach these various aspects successively, before comparing the results of the proposed methods of simulation with measures carried out on real monitored work. Graphical output are included to illustrate displacements, strains, pressures, stresses and failure mechanisms.

### 1.2 Literature review of finite elements analysis for excavation retained by walls stability

With the rapid development of urbanization, the focus on space development has gradually shifted underground, and a large number of excavations have therefore been constructed. Excavation inevitably influences the adjacent structure, which may lead to surface subsidence, building cracking, slope stability and retained walls. Therefore, excavation induced impact on the surroundings near the ground has attracted a considerable amount of attention [5–12]. Backfill on the soft soils behaviors differ under varying conditions, including hydrogeological conditions, brace form, and geometry, due to the complexity of the problem. The earth pressure acting on the retaining structure changes with time, while the soil strength reduces as a result of creep. Unreasonable design of the retaining structure of the foundation pit as well as the construction plan may lead to serious engineering accidents and cause great economic loss [13–16]. Therefore, it is extremely important to minimize excavation induced effects and control deformation for geotechnical structure interaction safety, affected by various factors [4,16]. The phenomenon mentioned above namely, the close relationship between the lateral displacement of the multi-layered soft soil retained by stell sheet pile walls and the changing soil behavior owing the spatial geometry and the time-space effect. Many theory have been proposed in litterature to control the lateral displacement of the retaining wall by adjusting calculation parameters in consideration of the staged construction [4,16,17]https://www.sciencedirect.com/topics/engineering/construction-process. The research developed by Kort [18] is focused on two analysis in the design of steel sheet piling (plastic design and oblique bending). He present three classical methos for plastic design of steel sheet pile walls (method of Brinch Hansen, Windels and Weiβenbach). These three methods focus on the geotechnical aspects of plastic design and assume an unlimited rotation capacity for the steel sheet piles. On the basis of the finding, Kort [18] develop a new design method to take oblique bending into account. From evaluation of the full-scale field test results, he as concluded that the behavior of the plastic hinge was as expected, meaning that the assumptions on which the design rules in ENV 1993–5. The analysis of the interactions between the retaining walls structure, the grounds and the neighbouring structuress requires to improve the techniques of numerical simulation, notably the finite element method applied to retaining structures [19]. In this arena, Lim [20] develop a novel strut-free earth retaining wall system for excavation in soft clay, referred to as the rigid and fixed diaphragm (RFD) wall retaining system. The RFD system is comprised of four main structures diaphragm walls, rib-walls, cross walls, and buttress walls and a complementary structure the cap-slab. The characteristics of the RFD system are: (1) the formation of a continuous earth retaining wall by constructing diaphragm walls along the circumference of the excavated zone; (2) the formation of a rigid and fixed retaining wall system by a series of rib-walls and cross walls; and (3) the formation of a rigid retaining wall by buttress walls and the cap-slab. The performance and mechanisms of the RFD system were investigated carefully through three-dimensional finite element analyses [20]. The results demonstrated that the system stiffness of the RFD system was a major factor controlling deformations induced by excavation.

Based on 18 excavation cases selected to study excavation behavior characteristics, the corner effect was found to exist, and according to a numerical analysis, the maximum lateral movement of the retaining structure was affected by the pit length [6]. Moreover, parametric studies were carried out, the results of which demonstrated that controlling parameters such as the horizontal displacement of the soil at the deep layer, ground surface settlement, and the supporting axial force exhibit obvious spatial distribution characteristics. In recent years, numerous pits with irregular geometries have been extensively used in a variety of practical engineering applications, due to the complicated environment surrounding pits and limited construction space [16]. The innovative prestressed support (IPS) earth retention system was proposed in order to be suitable for limited construction space. The IPS earth retention system has been applied in various excavations for its particular advantages, in that it can provide larger construction space and high security by means of prestressed wire tensioning to restrict wall deformation. In addition to the retaining structure factors, including support form, excavation geometry, wall stiffness, and diameter ratio, the excavation sequence plays a vital role in controlling the foundation pit deformation response. Layered soils and blocked excavation measures were taken, benefiting from the time-space effect theory, which control the deformation induced by soil resistance, so as to reduce the retaining wall deformation. There still exists uncertainty surrounding multi-layered and blocked excavation, making it is challenging for design engineer to get achieve improved understanding of retained stell sheet pile walls behavior under different excavation schemes. Subsequent use of the FE method in slope and retained walls stability analysis has added further confidence in the method. Coal slopes at Yallourn open-pit mine, Victoria, Australia was considered, forming the basis of a case study to demonstrate the applicability of the method. Huang [21] extended the use of strength reduction FEM to include the effects of unsaturated transient seepage and some primary numerical results concerning the stability of an earth dam under rapid drawdown have been presented. A stability analysis performed on the Yashigou earth dam in China was conducted by Huy [22]. Zoa [17] conducted a finite element analysis of a soft soil reinforced by an industrial nailing technique using a soft soil behavior model. He determined the main influential parameters. A numerical model termed as ISR3DNMM-GPS method has been developed by Yantao [23] for factor of safety calculation and 3D failure surface determination involved in a 3D slope stability analysis. In this proposed numerical model, an improved strength reduction technique (ISRT) which can avoid unreasonable plastic zones appearing in the deep area of a slope has been implemented into 3D numerical manifold method, and improved strength reduction based 3DNNM is developed. The study by Gholampour and Johari [24] presents a practical appoach for analysing the reliability of braced excavations in spatially varied unsaturated soils. To take full advantage of measured data, the random fields were conditionally simulated and implementedin finite element analysis. In this approach, the uncertainties involved in soil-structure interaction were taken into account by performing the analysis of perfectly smooth and perfectly rough inerfaces leading to upper and lower bounds on the full range of solutions [24]. Wong [25] gives a useful summary of potential sources of error in the FE modelling of slope and retained walls stability. Other important contribution in this area come from the work of Sloan [26] who published the papers concerning the advances in stability analysis that combine experimental and the limit theorems of classical plasticity with finite elements to give rigorous upper and lower bounds on the failure load. He proposed a new development, which incorporates pore water pressures in finite element limit analysis and proposed of the new techniques for stability solutions including foundations, anchors, slopes, excavations and tunnels. Tschuchnigg [27] carried out a comparative study concerning the strength reduction method and rigorous limit analyses which are based on collapse theorems of plasticity. An open-cut approach using steel-sheet piles and jet grouting piles for waterproofing was investigated by Han et al., [28] to resolve the problem that ordinary pipejacking equipment cannot cross areas with existing anchor cables in soft stratum. The case history of a pipe-jacking project of a sewage treatment plant in the Jinan East Railway Station area was investigated. The mechanical properties of steel-sheet piles, horizontal displacement of piles, and ground surface settlement in the anchor-cable crossing area were investigated based, on in situ observations. Numerical investigations were performed using the finite element method (FEM). The effects of existing anchor cables on the mechanical behaviors of retaining structures, deformation variation of the ground, and stability of the excavation were studied. The results indicate that the composite supporting structures of steel-sheet piles and jet grouting piles have a positive effect on waterproofing and deformation control in areas with existing anchor cables. When the steel-sheet pile touched the anchor cable during pile jacking, the compressive stress at the pile cap increased rapidly until it reached 62.8 MPa (the maximum pressure provided by the pile-pressing machine), which is twice the pressure under ordinary conditions. The maximum horizontal displacement of the retaining pile, increased linearly with the excavation depth. Existing anchor structures behind the excavation can restrain the deformation of the ground and retain the structure to a certain extent [28]. Johari and Kalantari [29] studied the stochastic framework with a random elasto-plastic finite element-based program coded in MATLAB to evaluate the reliability indices of individual failure modes with considering the inherent uncertainly of real site soil properties and unsaturated state. The sequential compounding method (SCM) was utilized to obtain the system reliability index by compounding the reliability indices of individual failure modes in the next step [29]. Numerical results showed that in all failure modes, considering unsaturated state not only increases the mean value of factor of safety (FS) but also decreases the related standard deviation, which can be counted as a goal of reliability analysis. Among the reliability indices of the components, the most critical one is attributed to the lateral displacement [29]. Lim and Ou [30] studied the stress state of soils during deep excavation, in relation to the determination of appropriate soil parameters for deformation analysis of a deep excavation case using the finite element method. Two well documented case histories of a deep excavation were utilized for the validation of the analysis procedure and the selection of soil stiffness parameters. Results from the Hardening Soil model showed that the out-of-plane stress has significance influences to the direction of soil effective stress path. Even though the effective stress path of soils adjacent to the diaphragm wall have undergone yield, but the characteristics of those soils are still dominated by the elastic behavior. Hence, the unloading/reloading parameters are predominant in a deformation analysis of an excavation case. The stability of the retaining system under seismic conditions is an important aspect of safe design in earthquake-prone areas. This stability is highly dependent on soil uncertainty and failure mode contribution. On the other hand, imaging the borehole data directly into the analysis section and ignoring the known data by using unconditional simulation can lead to unrealistic results. Reliability analysis of a real case study reveals that compared with the Unconditional Random Finite-Element Method (URFEM), utilizing the Conditional Random Finite-Element Method (CRFEM) helps improve the mean value of the Factor of Safety (FS) against all failure modes by 7%–30%, while reducing the related standard deviation by 12%–43%. The results of system reliability show that bending moment and lateral displacement are the fundamental mechanisms in the static and seismic states [31]. Based on excavation case history, Zhao et a*l*., [32] performed a numerical investigation using Plaxis FE code to reveal the crucial mechanichal behaviors of the retaining structure and soil layers as a result of different types of anchorage failures. The results of this investigation indicates that dangerous failure positions appear at the top of the pile wall or in the region near excavation bottom. The study performed by Johari et a*l*., [33] as an artificial intelligence method is used for developing a model to predict the discharge flow rate. The input parameters include the sheet pile height, upstream head, and hydraulic conductivity anisotropy ratio. A database including 1000 cases created from the Scaled Boundary Finite Element Method (SBFEM) for the seepage beneath sheet piles is employed to develop the model. The GEP-based model predictions are shown in reasonable agreement with the simulated data, which indicates the efficiency of the developed model. The results of the sensitivity analysis indicate that the upstream head is the most influential parameter in the discharge flow rate beneath the sheet piles.

According to the walls standard AFNOR [34] “a few thousandths of the total supported height (H) constitute a usual order of magnitude of the deflections of the walls for current structures”. In the literature, several databases collect measurements on instrumented deep excavations, and give reference points for horizontal displacements. The database proposed by Moorman [35], for the walls fixed by the struts or realized with the top-down method, shows typical values of the maximum deflections varying between 0.25%H for sandy soils and less than 0.5%H for stiff clays and layered soils. Moorman [36] indicates that horizontal displacements decreases by less than the increase in stiffness and that, in urban areas, the presence of buildings imposes additional support forces: it is therefore necessary to identify all overloads that may have an impact on the support. Marten [37] analyzed several deep excavations in France mainly supported by the walls and obtained a maximum horizontal deflection of less than 0.25% H. Clough and O’Rourke [38] studied a series of finite element numerical analyses. They defined two categories of works: those built in steep sands and clays and those built in soft to medium clays. A maximum horizontal displacement of 0.2%H is observed for the first category. Finno and Harahap [39], report that during a stumbled excavation in Chicago, the horizontal displacement is of the order of 1.4% of the headroom. Ou et al. [40–42], studied ten excavations in Taipei. Maximum horizontal displacement values between 0.2%H-0.5%H are observed. These values are generally larger than those suggested by Clough and O’Rourke [38]. Carder [43], analyzed excavations in the hardening soils of London. The maximum horizontal displacements were 0.125%H, 0.2%H or 0.4%H, depending on the stiffness of the support system. Wong et al. [44], analyzed excavations in Singapore, for which the maximum displacements depend on the thickness of the soft soils layers: with soft layers of less than 0.9H thick covering more stiff soil layers, the maximum horizontal displacement is 0.5%H. With layers of soft soil with a thickness of less than 0.6H, this value is 0.35%H. Long [45], presents 171 cases mainly in steep clays and sandy soils. Examination of the results led him to identify four categories of structures, depending on the thickness of the soft seams, the safety factor of the settlement of the excavation bottom, and the rigidity of the support system. It is interesting to note that for the stations of the « Grand Paris Express », Daktera [46,47], Nejjar [48] and Burlon [49], give maximum horizontal displacements of the order of 15 to 20 mm for excavation depths close to 30 m, which gives a significantly lower u_max_/H ratio than the values found in previous references, of the order of 0.07% H. Several other works [50–66] in this area have been carried out around the world both on deflections on the walls but also on the settlement at the bottom of the excavation and vertical displacements behind the wall.

The developments which follow in this paper, are inspired by these important contributions on this topic.

## 2. Methodology

**Fig 1:**
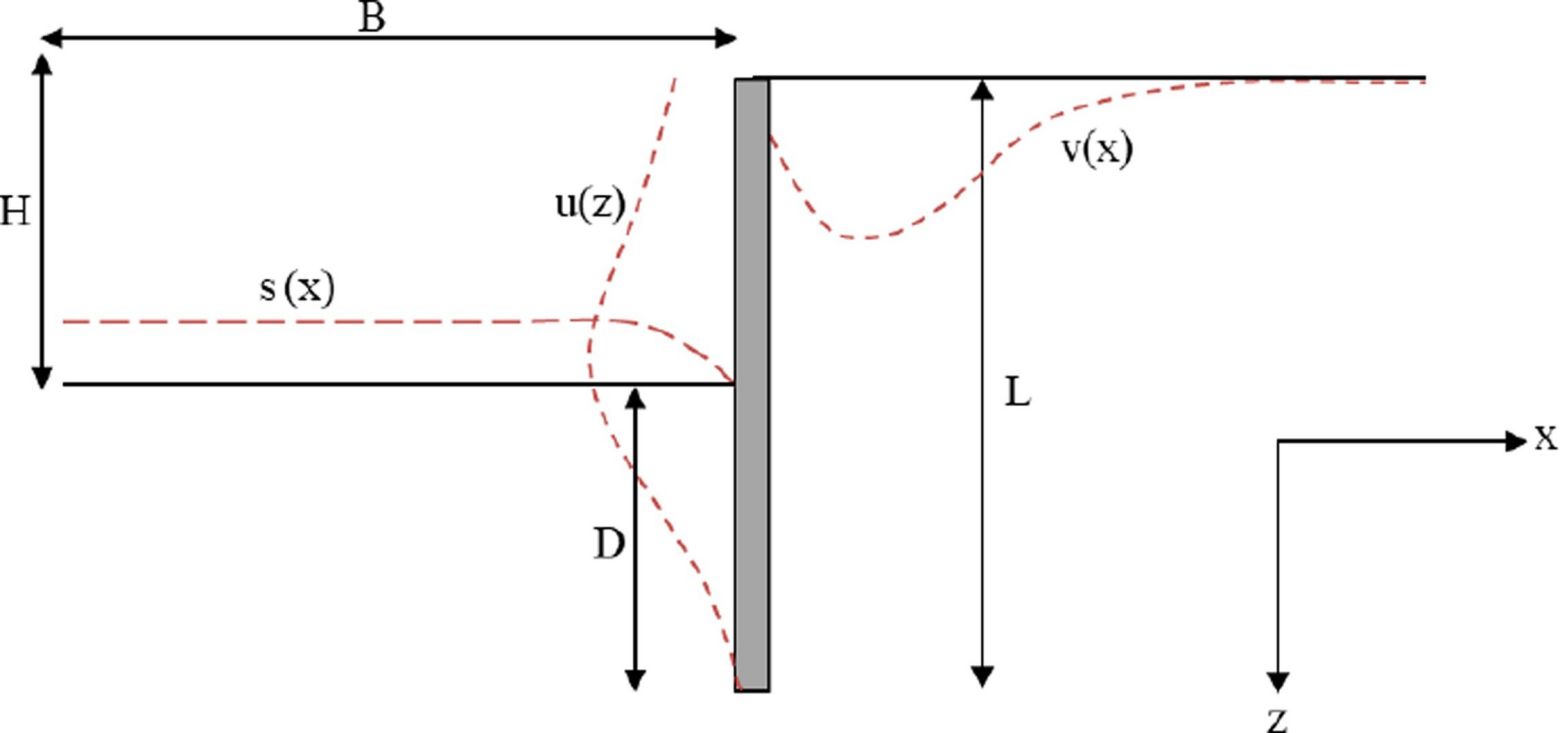
Geometric characteristics of an excavation retained by the wall.

In the case of deep excavations, particular interest is the horizontal deflection of the wall, the vertical displacement behind the wall, and the settlement of the excavation bottom. Fig 1 shows the notations used for the geometric characteristics of an excavation with a retaining wall with a head support (L: wall length, D: plug depth, H: headroom, B: half-width of the excavation, u(z) is the horizontal deflection of the wall, v(x) is the vertical displacement behind the wall and s(x) is the settlement of the excavation bottom).

### 2.1 Elasto-plastic model of the soil in the site

The first step of the proposed approach consists in characterizing on the one hand the behavior of the soil ground constituting the structures whose stability is sought to be assessed by choosing an elastoplastic law to represent them, and on the other hand in estimating in a relevant way a value deterministic for each of the parameters of the constitutive law, which associated with other variables, will constitute the input data of the Finite Element model. The choice of a constitutive law depends on the mechanism to be modeled, the data available for the structures, but above all on the precision sought and numerical considerations. We are interested in the mechanisms that can lead structures under extreme stresses to failure. However, to calculate the stability relating to the behavior of geotechnical structures by the Finite Element Method, the evaluation of the elastic deformations does not matter because it is the modelling of the appearance and the evolution of the unstabilized plastic strains which is important. Among the laws at our disposal and described in Mestat [67], we present here two classic behavior laws of the elastoplastic type: those of Mohr-Coulomb and Drucker-Prager. These two laws have the advantage of depending on few parameters directly from triaxial and oedometric tests. They allow a certain “economy” of data acquisition and management which are, in particular those concerning the mechanical characterization of materials, relatively few in number and not very varied on geotechnical projects.

**Fig 2:**
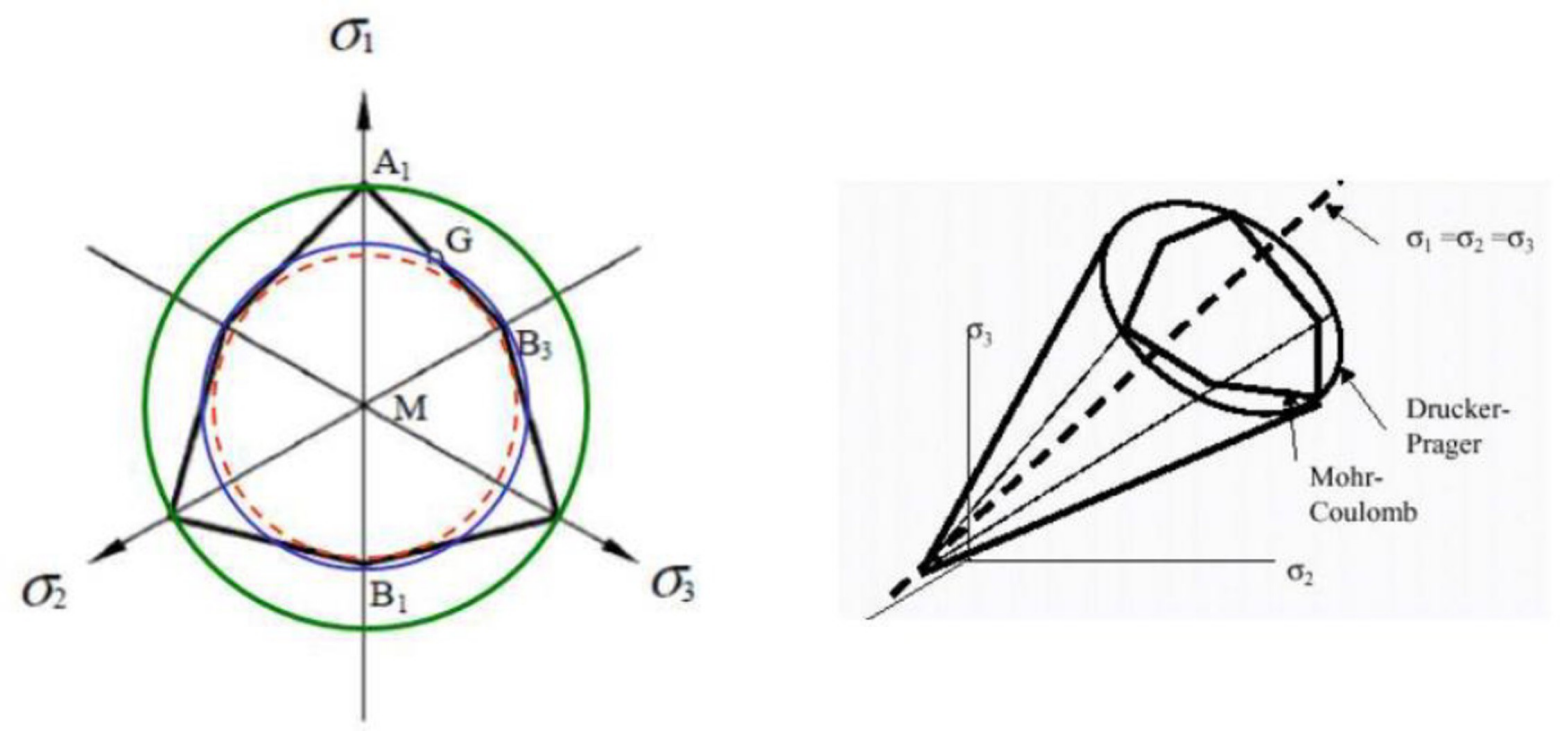
Mohr-Coulomb and Drucker-Prager yield criteria.

The Mohr-Coulomb criterion is the first that was proposed for soils [67]. It is used for long-term for frictional and cohesive soils. It is characterized by the relations:

$$\text{F}\left(\sigma_{\text{ij}}\right)=\left(\sigma_{1}-\sigma_{3}\right)-\left(\sigma_{1}+\sigma_{3}\right)\sin\;\varphi -2\;\text{c}\;\cos\;\varphi \leq 0$$ (1)

$$\text{G}\left(\sigma_{\text{ij}}\right)=\left(\sigma_{1}-\sigma_{3}\right)-\left(\sigma_{1}+\sigma_{3}\right)\sin\;\psi +{\text{c}}^{\text{ste}}$$ (2)

where c is the cohesion of the material, φ is the friction angle, and ψ is the dilatancy angle. σ_1_ and σ_3_ represent the principal stresses (σ_1_ ≥ σ_2_ ≥ σ_3_). In the principal stress space, the surface defined by the Mohr-Coulomb criterion is a hexagonal pyramid with axis (σ_1_ = σ_2_ = σ_3_) (Fig 2). Analogies are possible between the Mohr-Coulomb and Drucker-Prager criteria (Fig 2). Relations can be established between the parameters (α, γ, k) and (φ, ψ, c) in certain situations [67].

The Drucker-Prager criterion is written:

$$f\left(\sigma \right)=\sqrt{J_{2}}+\alpha I_{1}-K\leq 0$$ (3)

$$f\left(\sigma \right)=\sigma_{e}+\sin\;\alpha I_{1}-K'\leq 0$$ (4)

The correspondence between the Mohr-Coulomb criteria and those of Drucker-Prager are as follows [68]:

$$ALFA=ETA=\frac{2\sqrt{3}\sin\;\varphi }{9-\sin^{2}\;\varphi },GAMMA=\frac{2\sqrt{3}\sin\;\psi }{9-\sin^{2}\;\psi },K=KL=\frac{6\sqrt{3}c.\cos\;\varphi }{9-\sin^{2}\;\varphi }$$ (5)

Moreover, the choice of a criterion must also be guided by the possible numerical difficulties of implementing the criterion. Some constitutive laws, such as that of Mohr-Coulomb for example, have a failure criterion comprising singular edges (Fig 2), which can numerically result in convergence difficulties [68–73]. In view of the above, the Drucker-Prager model implemented in the Cast3M Finite Element code will be used in this paper to describe the elasto-plastic behavior of soft soils materials.

### 2.2 Stress and displacement in a soil structure interaction

The geostatic stress analysis is based on the existence of soil layers specified by the user during calculation. The normal stress in the *i*^*th*^ layer is calculated according to:

$$\sigma_{i}=\sum h_{i}.\gamma_{i}$$ (6)

If the layer is found below the ground water table, the unit weight of soil below the wate table is specified with the help of input parameters of the soil as follows:

$$\gamma_{su}=\gamma_{sat}-\gamma_{w}\;\text{or}\;\gamma_{su}=\left(1-n\right)\left(\gamma_{s}-\gamma_{w}\right)$$ (7)

where h_i_: thickness of the i^*th*^ layer, γ_i_: unit weight of soil, γ_sat_: saturated unit weight of soil, γ_w_: unit weight of water, n: porosity, γ_s_: specific weight of soil.

The elastic vertical displacement can be checked by the formula given by Mestat et a*l*., [65]:

$$s=\frac{\left(1+\nu \right)\left(1-2\nu \right)}{2E\left(1-\nu \right)}\left(\gamma y^{2}-2\gamma Hy-2Py\right)$$ (8)

where: E: Young modulus of the soil, ν: Poisson ratio of the soil, y: vertical axis, H: height of de ground and P: vertical stress applied to the top of the soil.

The maximum bending moment (kN.m/m) on each wall can be obtained from the horizontal tensile stresses coming from these walls using the formula:

$$M=\frac{b.{\left(t^{eq}\right)}^{2}}{6}.\sigma_{x}$$ (9)

where: b: sheet pile width, M: bending moment, σ_*x*_: tensile stress in the wall, t^eq^: tickness of the wall (Table 2).

### 2.3 Earth pressure at rest in the model

Earth pressure at rest is the horizontal pressure acting on the rigid structure. It is usually assumed in cases, when it is necessary to minimize the lateral and horizontal deformation of the sheeted soil (e.g. when laterally supporting a structure in the excavation pit up to depth below the current foundation or in general when casing soil with structures sensitive to nonuniform settlement), or when structures loaded by earth pressures are due to some technological reasons extremely rigid and do not allow for deformation in the direction of load necessary to mobilize the active earth pressure. Earth pressure at rest is given by Jaky [63]:

$$\sigma_{h}=K_{0}.\sigma_{v}^{'}\;\text{and}\;K_{0}=1-\sin\varphi^{'}$$ (10)

### 2.4 Active and passive earth pressure in the model

**Fig 3:**
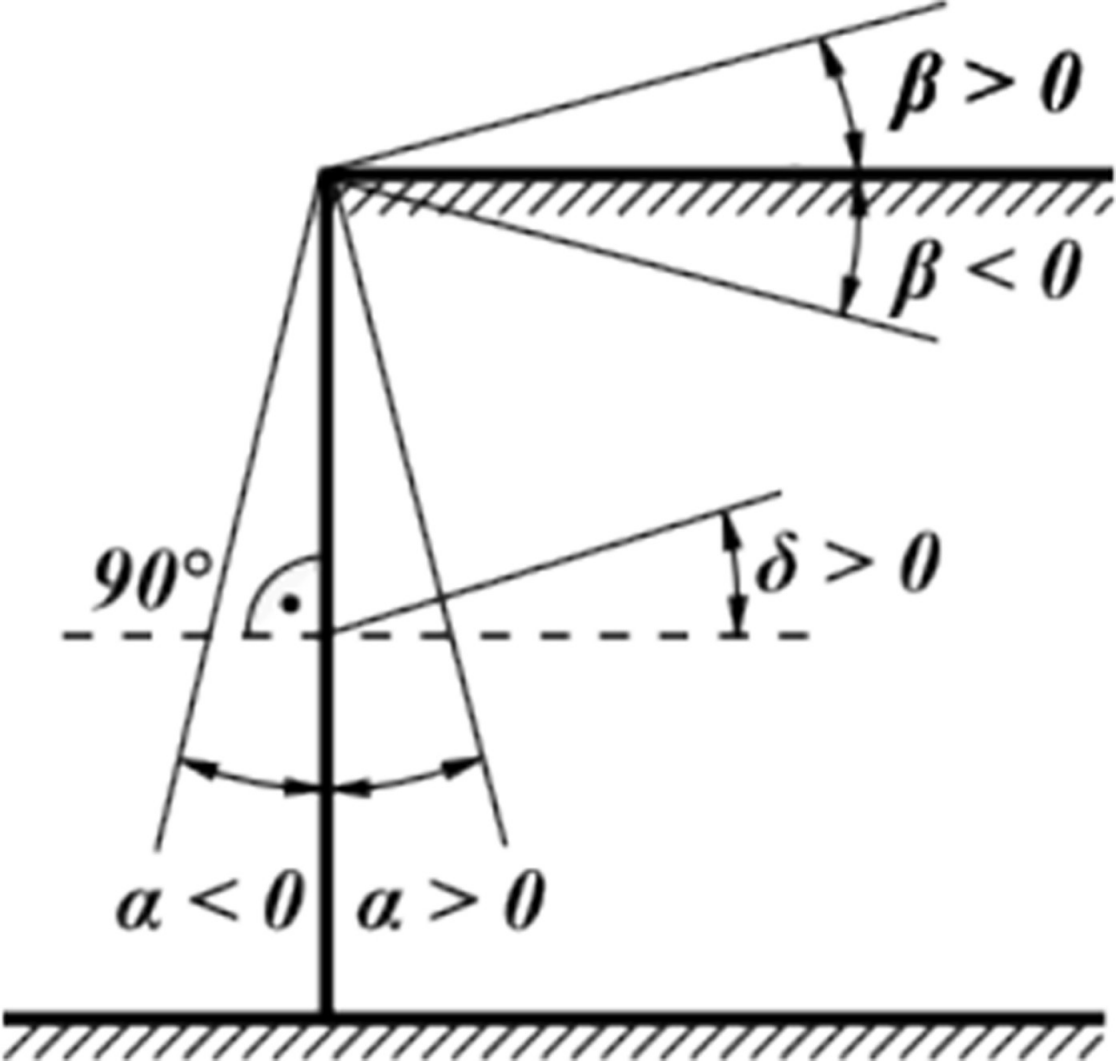
Sign convention for calculation of earth pressures.

Active earth pressure is the smallest limiting lateral pressure developed at the onset of shear failure by wall moving away from the soil in the direction of the acting earth pressure. Active and passive earth pressure is given by the following formula (Fig 3) of the Mazindrani Theory (Rankine) [64]:

$$\sigma_{a}=K_{a}.\sigma_{z}=\gamma .z.K_{a}^{'}.\cos\;\beta$$ (11)

$$K_{a}^{'}=\frac{1}{\cos^{2}\;\varphi }\left[\begin{array}{l} 2\cos^{2}\;\beta +2\left(\frac{c}{\gamma .z}\right)\cos\;\varphi .\sin\;\varphi -\\ \sqrt{4\cos^{2}\;\beta \left(\cos^{2}\;\beta -\cos^{2}\;\varphi \right)+4{\left(\frac{c}{\gamma z}\right)}^{2}\cos^{2}\;\varphi +8\left(\frac{c}{\gamma z}\right)\cos^{2}\;\beta .\sin\;\varphi .\cos\;\varphi } \end{array}\right]-1$$ (12)

$$\sigma_{p}=K_{p}.\sigma_{z}=\gamma .z.K_{p}^{'}.\cos\;\beta$$

$$K_{p}^{'}=\frac{1}{\cos^{2}\;\varphi }\left[\begin{array}{l} 2\cos^{2}\;\beta +2\left(\frac{c}{\gamma .z}\right)\cos\;\varphi .\sin\;\varphi +\\ \sqrt{4\cos^{2}\;\beta \left(\cos^{2}\;\beta -\cos^{2}\;\varphi \right)+4{\left(\frac{c}{\gamma z}\right)}^{2}\cos^{2}\;\varphi +8\left(\frac{c}{\gamma z}\right)\cos^{2}\;\beta .\sin\;\varphi .\cos\;\varphi } \end{array}\right]-1$$ (13)

where: σ_z_: vertical geostatic stress, K_a_: coefficient of active earth pressure due to Rankine, K_p_: coefficient of passive earth pressure due to Rankine, β: slope inclination (Fig 3), γ: unit weight of soil, z: assumed depth, K_a_’ coefficient of active earth pressure due to Mazindrani, K_p_’: coefficient of passive earth pressure due to Mazindrani, φ: angle of internal friction of soil and c: cohesion of soil.

### 2.5 Horizontal modulus of subsoil reaction

Based on the measurements on sheeting structures in different soils and computation of a displacement of the structure needed to mobilize the limit value of active and passive pressure Chadeisson [59], Monnet [60] and Dhouib [61] derived expression for the determination of the active and passive horizontal modulus of subsoil reaction in the form:

$$k_{ha}=\beta_{a}.{\left[30.E.I.{\left(\frac{K_{a}.\gamma }{0.015}\right)}^{4}\right]}^{0,2}+A_{a}.c'.\frac{\tanh\left(\frac{c'}{30}\right)}{0.015}\;\text{and}\;\beta_{a}=\left(\frac{K_{0}}{K_{a}}-1\right)$$ (14)

$$k_{hp}=\beta_{p}.{\left[\frac{120}{11}.E.I.{\left(\frac{K_{p}.\gamma }{0.015}\right)}^{4}\right]}^{0,2}+A_{p}.c'.\frac{\tanh\left(\frac{c'}{30}\right)}{0.015}\;\text{and}\;\beta_{p}=\left(1-\frac{K_{0}}{K_{p}}\right)$$ (15)

where: EI: bending stiffness of the structure [kNm^2^/m], γ: unit weight of soil [kN/m^3^], K_a_: coefficient of active earth pressure, K_p_: coefficient of passive earth pressure [–], K_0_: coefficient of earth pressure at rest from Jaky formula [63], c’: effective cohesion of soil [kPa] and A_c_ /A_p_: coefficient of influence of cohesion (1–15).

## 3. Numerical modelling of the test site

**Fig 4:**
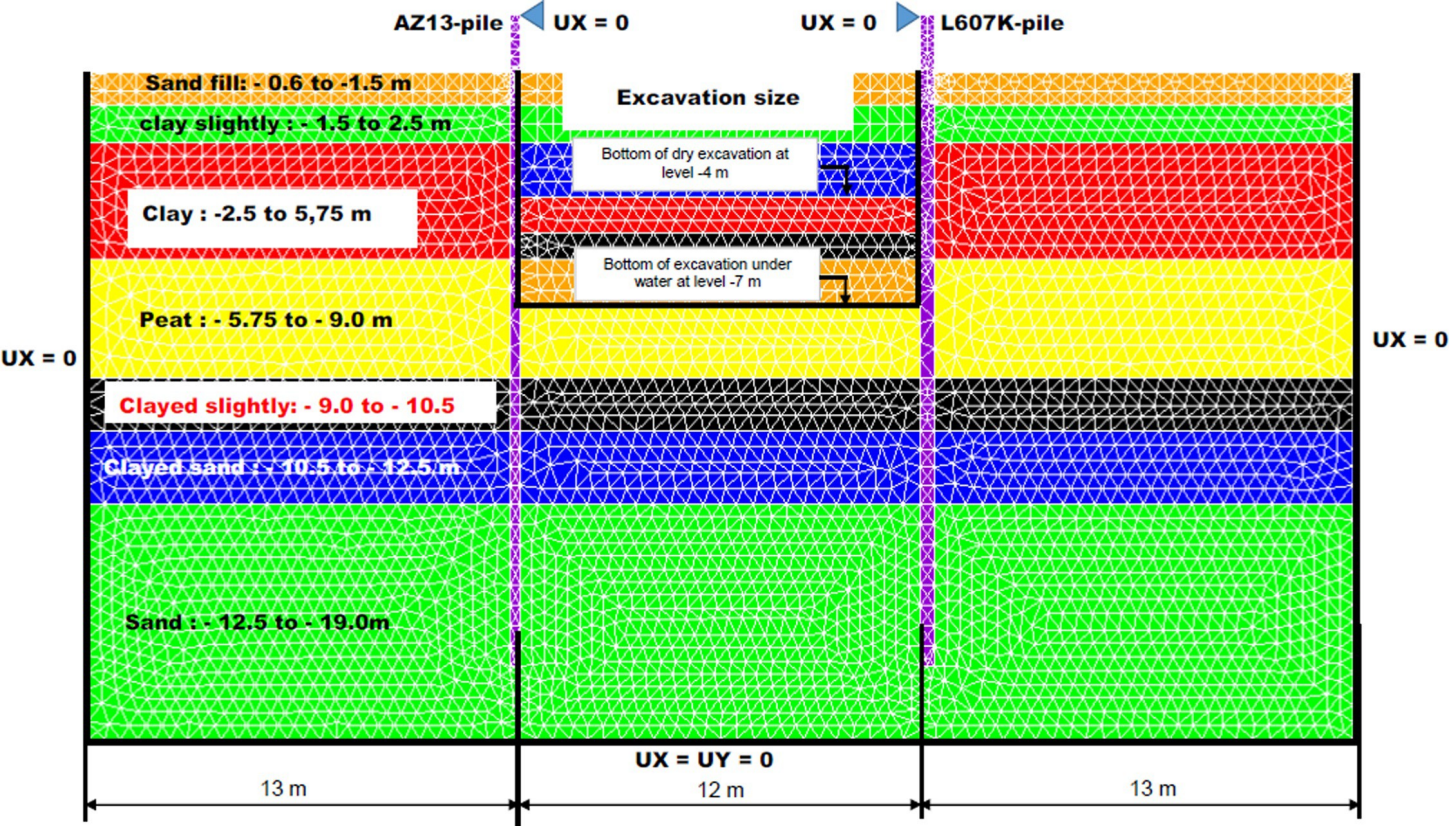
Detailed view of the 2D mesh and soil clusters of the deep excavation in submerged multi-layered soft soils retained by two stell sheet pile walls.

The test site was situated near Pernis, which is a suburb west of Rotterdam (Netherland), and formed part of the construction site for metro line in Rotterdam. In the Pernisserpart a small area of land was available, about 20 x 50 metre, where the field test could be carried out. The struts for the north and south test walls were designed in a such way that both test walls could act as independently as possible and that influence from other sheet piles was minimised. The struts and the walings were constructed as hinged connections with sufficient rotation capacity betwen the sheet pile walls and the frames [18]. The horizontal displacement curves of the test wall were determined in the site from inclinometer measurements [18]. The numerical modelling of the deep excavation in submerged multi-layered soft soil retained by stell sheet pile walls structures is made in 2D, plane strains from the Finite Elements Cast3M [74] calculation code with triangular elements (7910 element) with 3 nodes (TRI3). Fig 4 shows the 2D mesh view of the model and the borehole log with the altimetry of the different soils. The boundary conditions of the model are standard, i.e. blocking of all displacements at the bottom of the model (Ux = Uy = 0), blocking of horizontal displacements on the left and right bound of the model (Ux = 0) and blocking horizontal displacement at the top of the wall on the excavation size for the phases 1 to 8. Zero groundwater flow is also fixed on the stell sheet pile walls and the bottom, left and right bound of the model.

In this paper, the forces generated by the self-weight of the soil are computed using a standard gravity ‘turnon’ procedure involving integrals over each element of the form:

$$p^{\left(e\right)}=\gamma \int_{V^{e}}N^{T}d\left(vol\right)$$ (16)

where N values are the shape functions of the element and the superscript *e* refers to the element number. This integral evaluates the area of each element, multiplies by the total unit weight of the soil and distributes the net vertical force consistently to all the nodes [4,75–80]. These element forces are assembled into a global gravity force vector that is applied to the FE mesh in order to generate the initial stress state of the problem.

### 3.1 Materials model

In Table 1, the characteristics of the AZ13 and the L607K stell sheet piles are given. The struts is made of metal beams of high inertia; they are therefore assumed to be infinitely stiff. The simulation of the struts supports was made by imposing a zero horizontal displacement for the two points of support (see Fig 4). Sheet pile walls are modeled by massive elements. The material is assumed to be isotropic linear elastic; Young E’s modulus is calculated assuming the conservation of bending stiffness. The inertia of AZ13 sheet pile is evaluated at 19700 cm^4^/m, which leads to an inertia product equal to 41370 kNm^2^. The stell sheet piles AZ13 and L607K were modelled as retaining walls of uniform thickness, *t* = 30 cm and *t* = 44 cm, respectively, of equivalent modulus of elasticity equal to $E_{AZ13}^{eq}=18467.3MPa$ MPa and $E_{L607K}^{eq}=13574.6MPa$. The equivalent modulus is given by $E^{eq}=\frac{EI}{I^{eq}}$where I^eq^ is the equivalent inertia of the uniform wall section considered (Table 2). The explanations provided for Table 1 are: A—sheet pile area per metre, I—sheet pile inertia per metre, W—sheet pile inertia modulus per metre, EI—bending stiffness per metre, M—mass per square metre, b—sheet pile width, t—sheet pile right-of-way height.

**Table 1:** Structural properties of the AZ13-pile and the L607K-pile.

Pile	A (cm^2/^m)	I (cm^4^/m)	W (cm^3^/m)	EI_yy_ (kNm^2^/m)	M (kg/m^2^)	b (cm)	t (cm)	t_f_ (mm)	c (mm)
AZ13	137	19700	1300	41370	107	67	30.3	9.5	-
L607K	244	70030	3220	147260	192	60	43.5	-	146

A—sheet pile area per metre, I—sheet pile inertia per metre, W—sheet pile inertia modulus per metre, EI—bending stiffness per metre, M—mass per square metre, b—sheet pile width, t—sheet pile right-of-way height.

**Table 2:** Mechanical properties of the AZ13-pile and the L607K-pile used for numerical modelling.

Pile	I^eq^ (cm^4^/m)	EI_yy_ (kNm^2^/m)	b (cm)	t (cm)	t^eq^ (cm)	E^eq^ (MPa)	Poisson ratio	γ (kN/m^3^)	Type of model	Type of behavior
AZ13	225000	41370	67	30.3	30	18467.3	0.2	24	Linear elastic	Non porous
L607K	710000	147260	60	43.5	44	13574.6	0.2	24	Linear elastic	Non porous

All geotechnical parameters characterizing the different soil layers constituting the model were determined in the laboratory by consolidated drained triaxial tests and by conventional oedometric tests from samples collected in the field. These are mainly soft soils with very low moduli of deformation. The mechanical and hydraulic parameters used are collected in Table 3. The dilatance angle is given by the following formula:

$$\left\{\begin{array}{c} \psi =0=>\varphi \leq 30\\ \psi =\varphi -30=>\varphi \geq 30 \end{array}\right.$$ (17)

**Table 3:** Soft soil properties of the model.

Parameter	Symbol	Sand	Clayed sand	Clayed slightly	Peat	Clay	Sand fill	Unit
Material model	Model	Mohr-Coulomb	Mohr-Coulomb	Mohr-Coulomb	Mohr-Coulomb	Mohr-Coulomb	Mohr-Coulomb	-
Type of behaviour	Type	Drained	Drained	Drained	Drained	Drained	Drained	-
Unit weight	γ’	10	6.34	3.9	2.3	6.75	17	kN/m^3^
Permeability x10^-9^	k_v_	0.3	0.3	0.3	1.0	0.6	10000	m/s
Young’s modulus	E_ref_	26.10^3^	7.32.10^3^	2.92.10^3^	2.34.10^3^	5.33.10^3^	17.3.10^3^	kN/m^2^
Poisson’s ratio	ν	0.3	0.2	0.2	0.2	0.2	0.3	-
Cohesion	c’	11.5	15.05	8.7	14.9	17.2	5.0	kN/m^2^
Friction angle	φ’	38.3	16.83	16.7	18.37	18.15	35.0	°
Dilatancy angle	ψ	8.3	0	0	0	0	5	°

Drucker-Prager model [81], ideal elasto-plasticity without hardening or softening, they constant stiffness parameters are adjusted of strength parameters to the average of Mohr-Coulomb compression and tension. Apart from unit weight of soil γ, Young Modulus E and Poisson’s ratio, this model requires 8 additional parameters, some of which are deduced from the Mohr-Coulomb model for its use in the F.E Cast3M code. Its other parameters are as follows:

$$ALFA=ETA=\frac{2\sqrt{3}\sin\;\varphi }{9-\sin^{2}\;\varphi }$$ (18)

$$BETA=MU=DELTA=\sqrt{\frac{2}{3}}$$ (19)

$$K=KL=\frac{6\sqrt{3}c.\cos\;\varphi }{9-\sin^{2}\;\varphi }$$ (20)

$$GAMMA=\frac{2\sqrt{3}\sin\;\psi }{9-\sin^{2}\;\psi }$$ (21)

In our computations on Cast3M, we have used a variable FCYS (FaCtor Yield Surface) for the built in Drucker-Prager plasticity model. In order to adjust the Mohr-Coulomb strength parameters to the circular cone of the Drucker-Prager failure surface (see Fig 2), different strategies are known. The factor FCYS regulates the adjustment [82]. The value of 0.0 is tension, 1.0 is compression, 0.5 is their average. Other values would mean a weighted result and values above 1.0 use the surface equality approach. Values below 0.0 default to 1.0 and the compression adjustment. In this paper FCYS is fixed at 0.5. Table 3 presents the soils properties of the model. $BETA=MU=DELTA=\sqrt{\frac{2}{3}}$ is implementation specific for Drucker-Prager model in Cast3M.

### 3.2. Modelling of the excavation procedure

To know the contribution of each phase to the global stability, the displacements are calculated. The solution strategy is based by treatment of the problem in 2-D plane strains (geometry and mesh), the definition of soils and retaining walls and the definition of all external loads (self-weight and charges) and the implementation of the excavation phases by stepwise imposition of loads [83]. The following staged constructions are analyzed in Cast3M: the phase 0 is based on the application of geostatic stress state in the sand fill (stress adjustment or gravity loading) to– 0.65 to—1.5 m. In phases 1.1 (free displacement at the top of the walls) and 1.2, the dry excavation to– 4.0 m is realized. The fill with water is observed in phase 2 to -1.5 m. In phase 3, the excavation under water to– 7.0 m is realized. The lowering water level to– 5.0 m is made in phase 4. The fill with water is observed in phase 5 to -1.5 m. The construction of sand backfill behind AZ13 stell sheet pile wall is realized in phase 6. The lowering water level to– 5.0 m is made in phase 7 and the long-term performance is analyzed in phase 8. In phase 9, blocking of horizontal displacements of the walls at 5.75m depth from the ground surface in the excavation area was carried out (2^nd^ bed of struts).

## 4. Drucker-Prager elasto-plastic modelling in staged excavation

In this part, the stresses and displacements are calculated for each excavation/construction stage. The “turn-on” procedure is described clearly according to the following calculation steps: After generation of the mesh of the global model, the calculation of the elastic phase is carried out in order to obtain the stresses and the elastic displacements in the structure. The elastoplastic calculation is carried out PASAPAS in the code Cast3M. Each excavation/construction stage of the structure corresponds to a PASAPAS calculation phase in the Cast3M code.

### 4.1. Phase 0: Ground and sand fill

**Fig 5:**
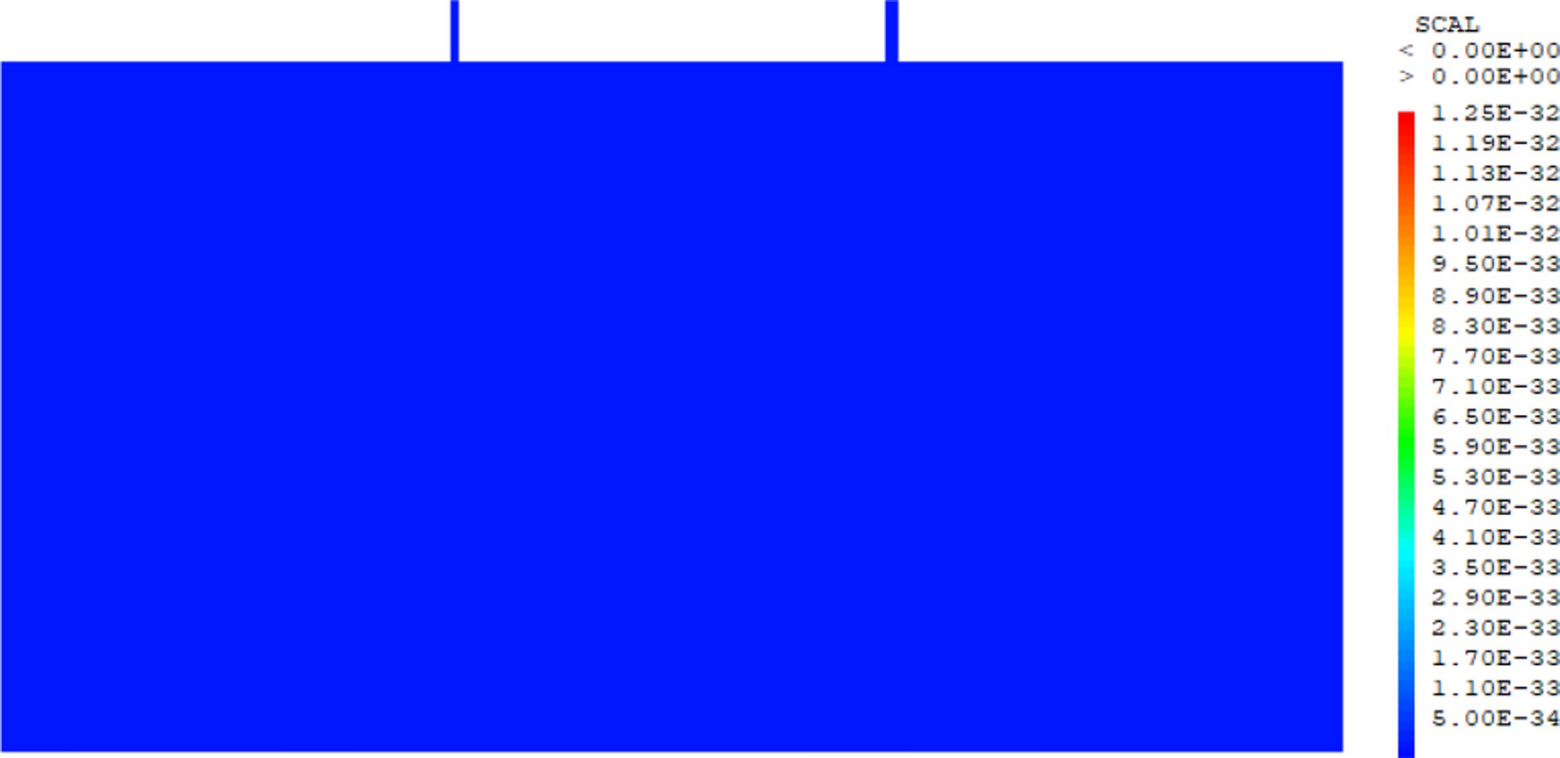
Shadings of the total displacements: U (m) of the model.

**Fig 6:**
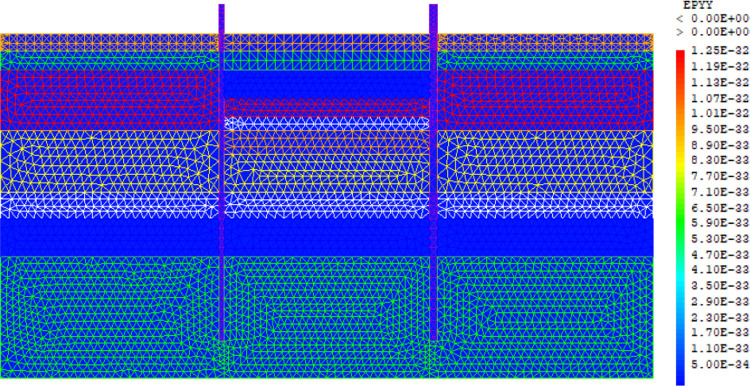
Shadings of the vertical strains of the model.

**Fig 7:**
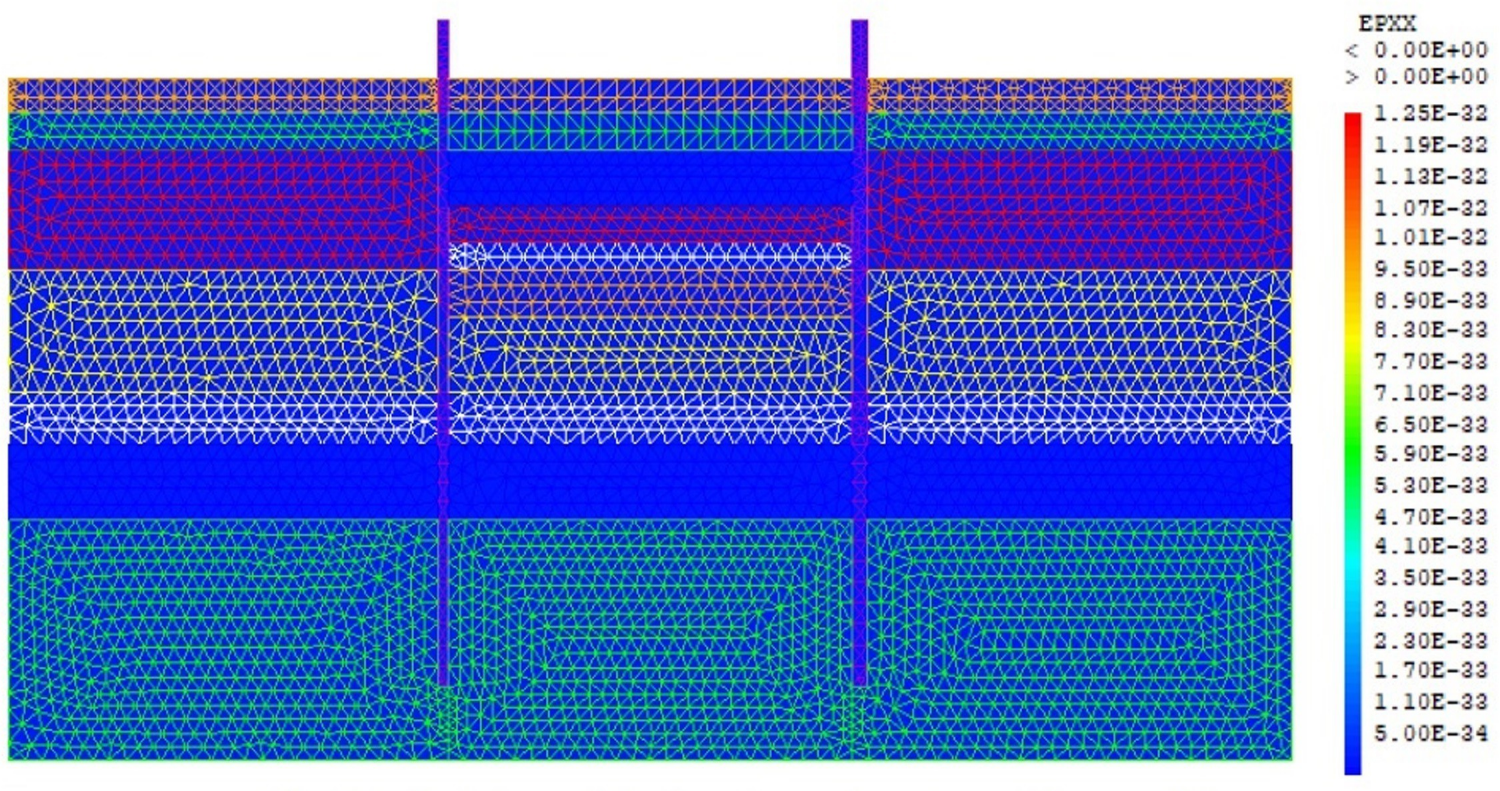
Shadings of the horizontal strains of the model.

**Fig 8:**
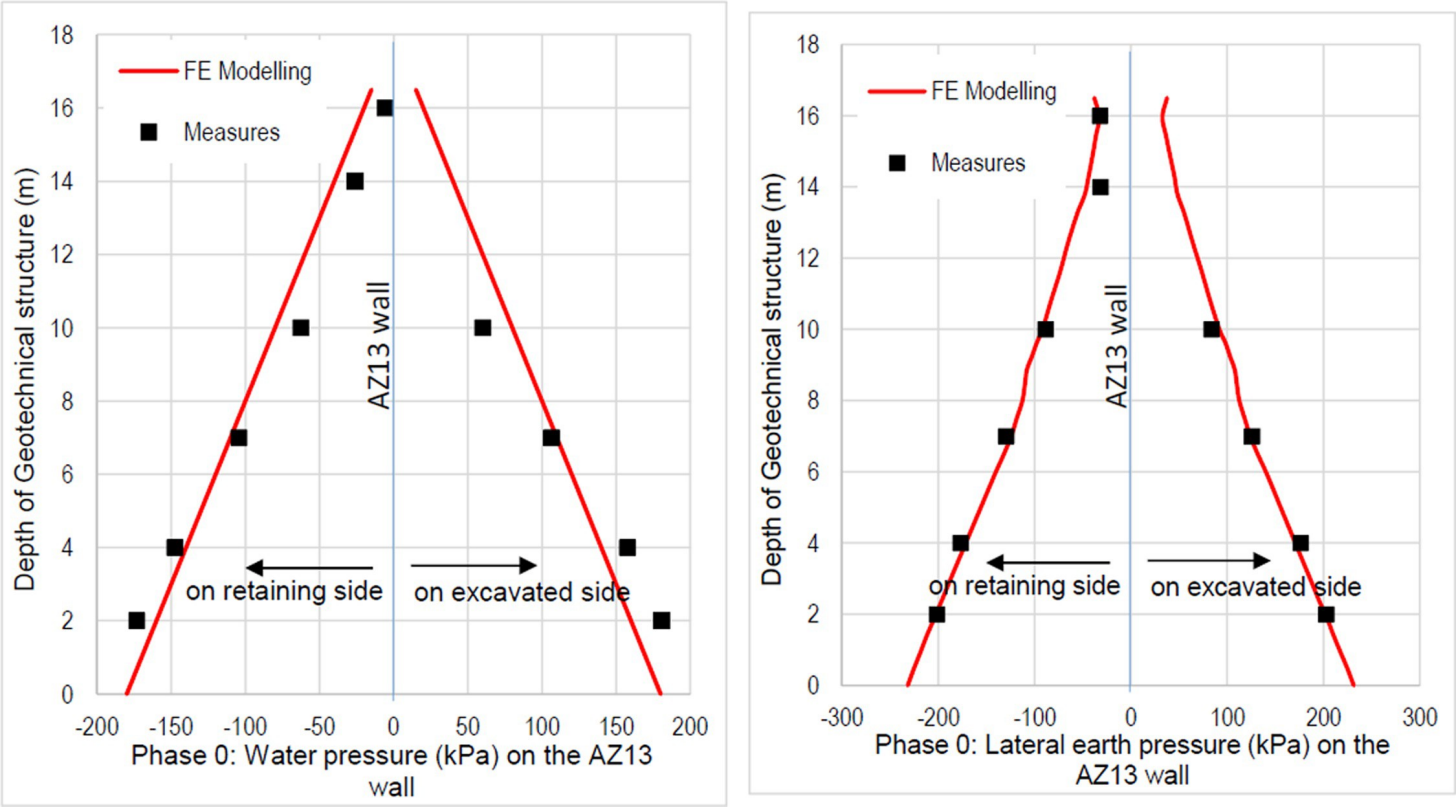
**a)** FE modelling vs. measurement results of the water pressure for the AZ13 wall; **b)** FE modelling vs. measurement results of the lateral earth pressure for the AZ13 wall.

The purpose of the elasto-plastic calculation carried out is to obtain the stresses and displacements throughout the model. The entire soil ground is built in a single phase. The gravity load are applied on the model. The importance of this phase is to assess the relevance of the modelling assumptions taken (walls, soil behavior, 2D plane strains, …). Figs 5–8 present the displacements, the strains, the water pressure and lateral earth pressure. The water and lateral earth pressure obtained by FE modelling are compared by those from measurements [18,19]. The total displacements and strains are of the order of 0.00 cm on the surface of the sand backfill beacause settlements were initialized to zero at this phase. The validation of the FE modelling is done by comparing the results of the model with those of on-site measurements. Fig 8 shows the results of water pressures and lateral earth pressures around the AZ13 wall. A perfect concordance is observed (FE results vs. measurement results), thus validating the assumptions of the FE modelling taken.

### 4.2. Phase 1: Dry excavation to– 4.0 m

**Fig 9:**
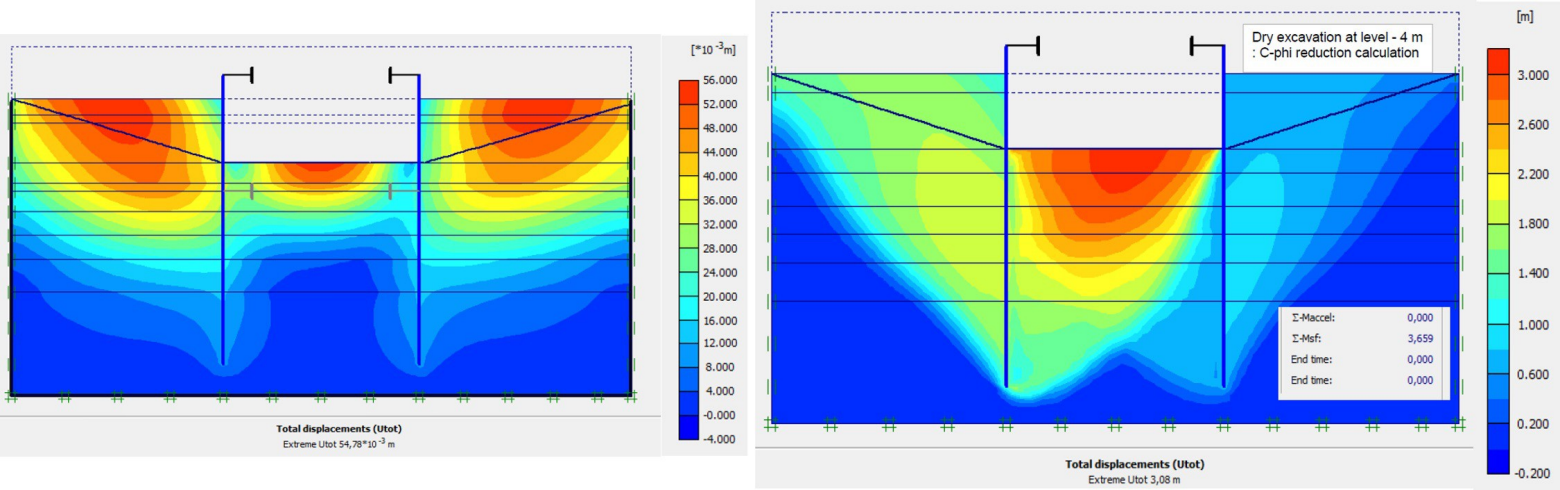
**a)** Shadings of the total displacements (m) of the model in phase 1.2; **b)** Shadings of the total displacements (m) of the model in phase 1 (c-phi reduction calculation—Factor of safety: 3.66).

**Fig 10:**
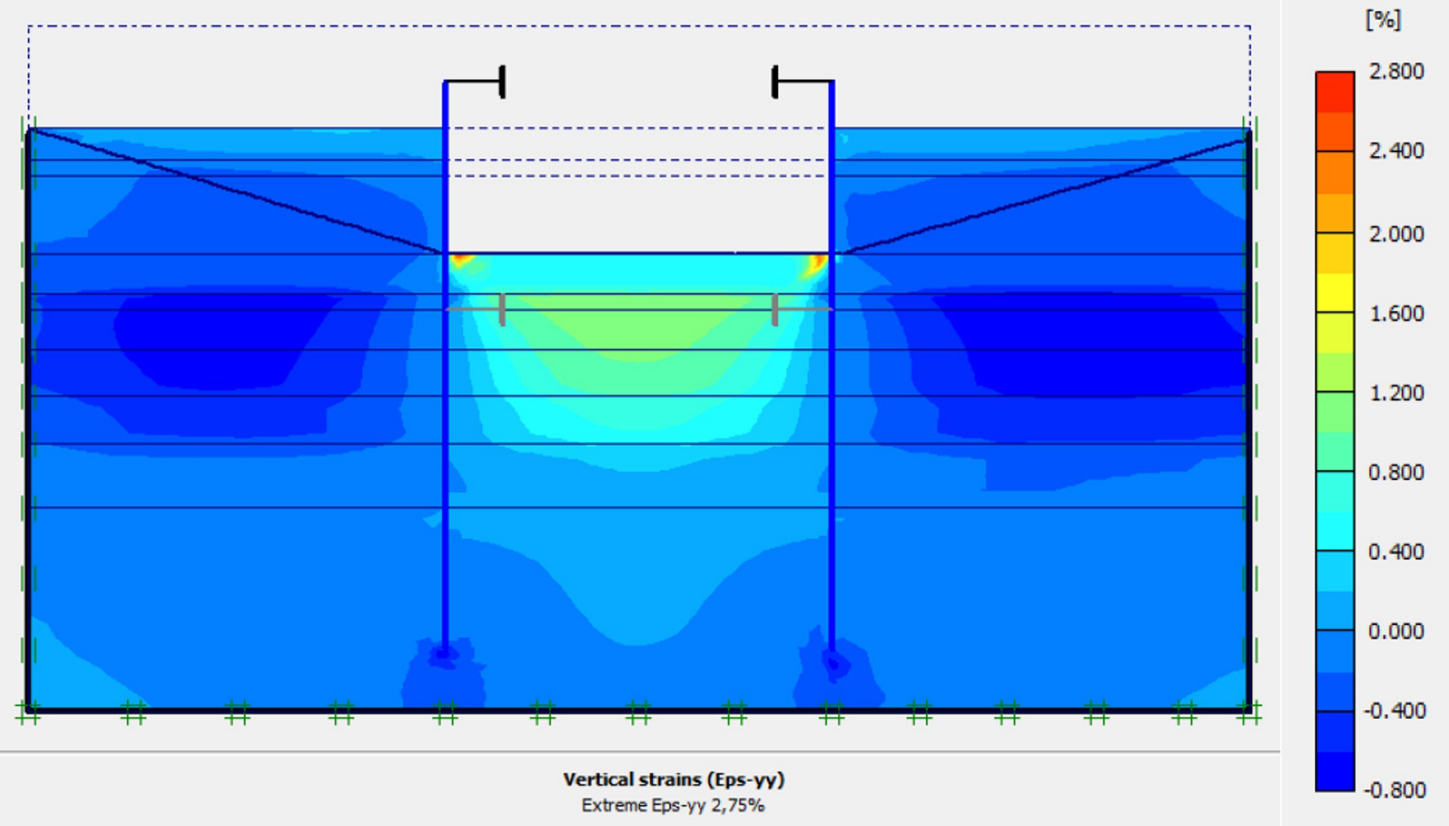
Shadings of the vertical plastic strains of the model in phase 1.2.

**Fig 11:**
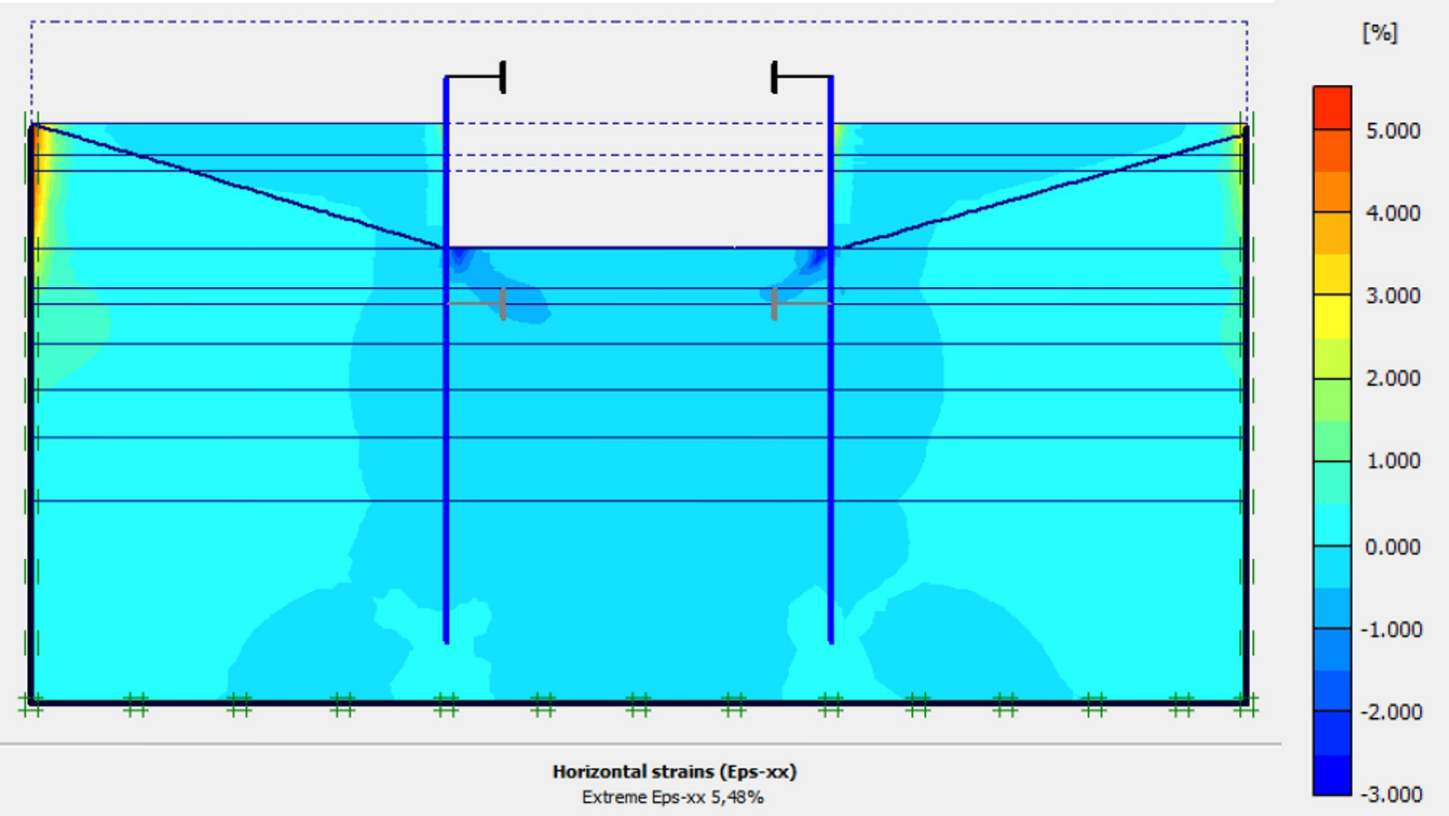
Shadings of the horizontal plastic strains of the model in phase 1.2.

**Fig 12:**
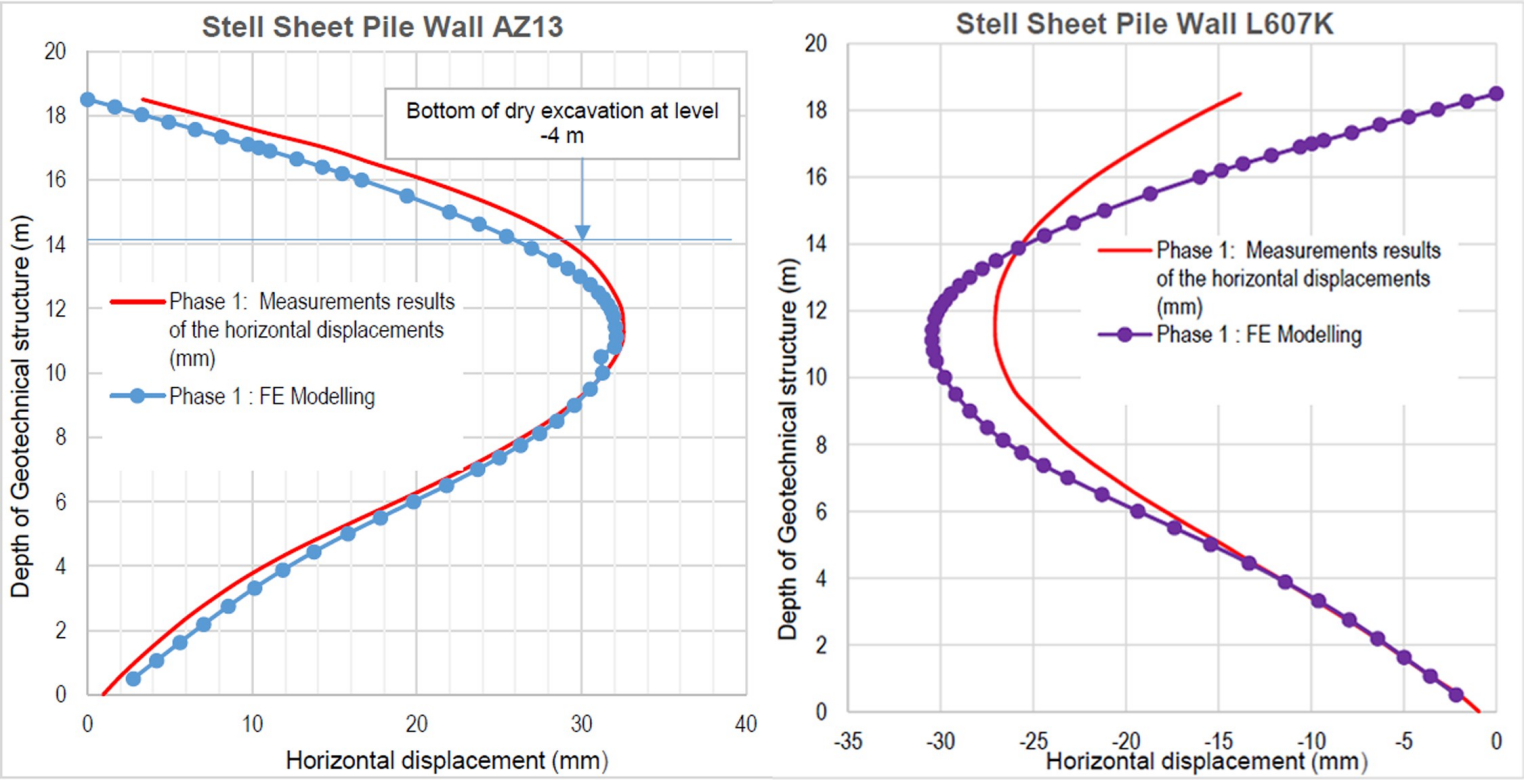
**a)** Horizontal displacements in the AZ13 stell sheet pile wall in phase 1.2; **b)** Horizontal displacements in the L607K stell sheet pile wall in phase 1.2.

The numerical modellings were carried out jointly with Cast3M and Plaxis finite element codes. This phase is based on the dry excavation of geostatic stress state in the ground. The excavation of the soft soils between the two walls induces a total displacement of 5.23 cm out of the excavation and about 5.45 cm inside the excavation (lifting). Figs 9A–11 present the total displacements, plastic strains and vertical stress (in phase 1.2) of the model from elasto-plastic analysis. A perfect consistency is observed on the horizontal displacements within the excavated zone on the walls AZ13 and L607K (Fig 12). The difference between the results of calculations and measurements remains small.

In the stability calculations, the strength reduction method has been used in Plaxis software. C-phi reduction is an option available in Plaxis [84] to compute safety factors. In the c-phi reduction approach, the strength parameters tanφ and c (cohesion) of the soil are successively reduced until failure of the structure occurs. The total multiplier **Σ***Msf* is used to define the value of the soil strength parameters at a given stage in the analysis (Fig 9B):

$$\Sigma Msf=\tan\left(\varphi_{\text{input}}^{'}\right)/\tan\left(\varphi_{\text{reduced}}^{'}\right)={\text{c}}_{\text{input}}^{'}/{\text{c}}_{\text{reduced}.}^{'}$$ (22)

SF=availablestrength/strengthatfailure=valueofΣMsfatfailure. (23)

### 4.3. Phase 2: Fill with water to -1.5 m

**Fig 13:**
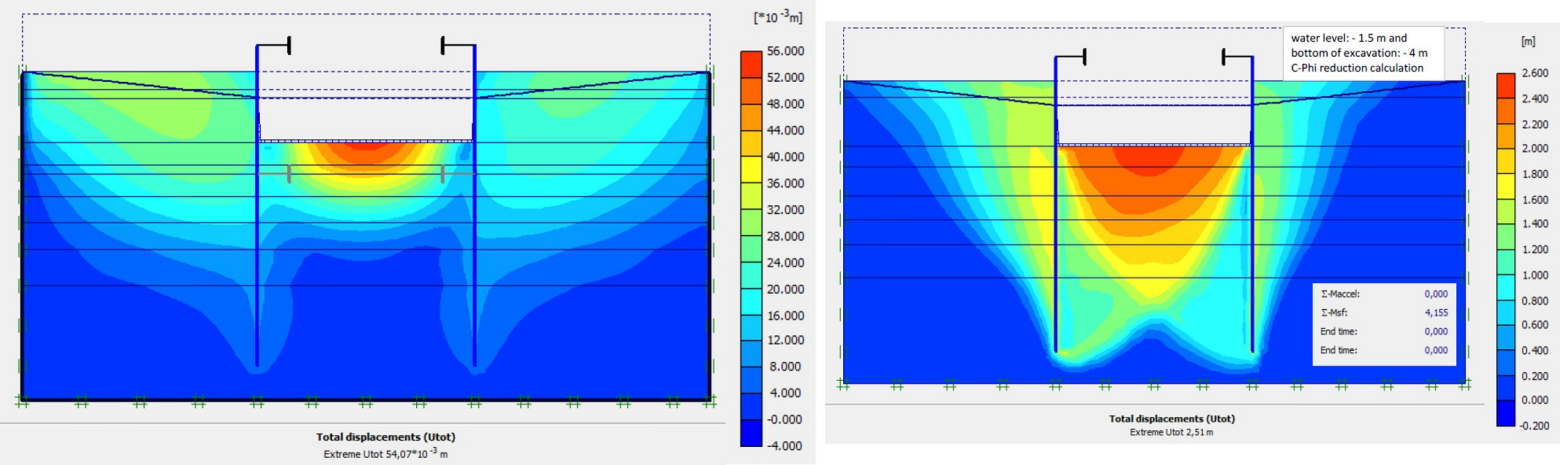
**a)** Shadings of the total displacements (m) of the model in phase 2; **b)** Shadings of the total displacements (m) of the model in phase 2 (c-phi reduction calculation—Factor of safety: 4.15).

**Fig 14:**
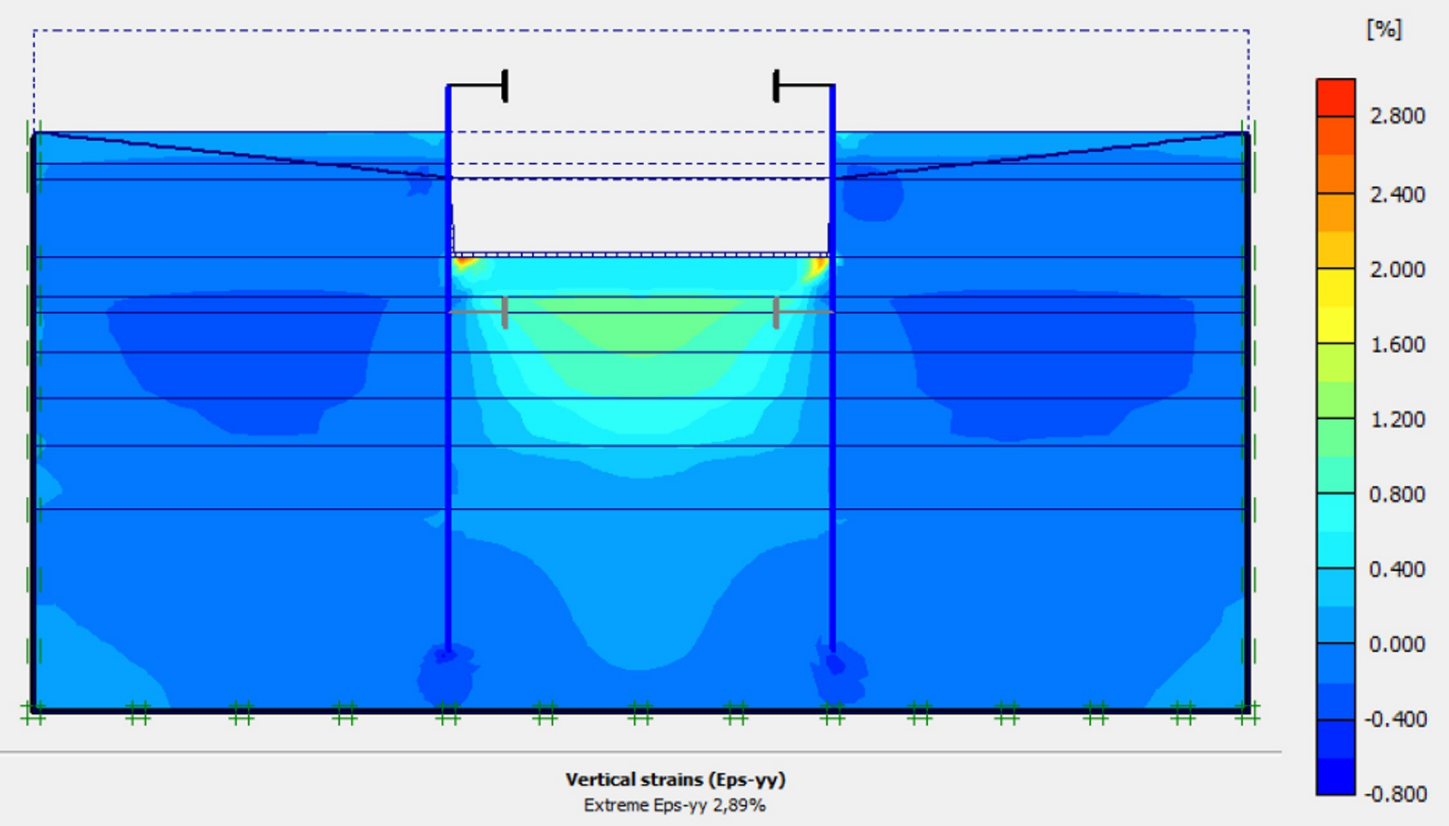
Shadings of the vertical plastic strains of the model in phase 2.

**Fig 15:**
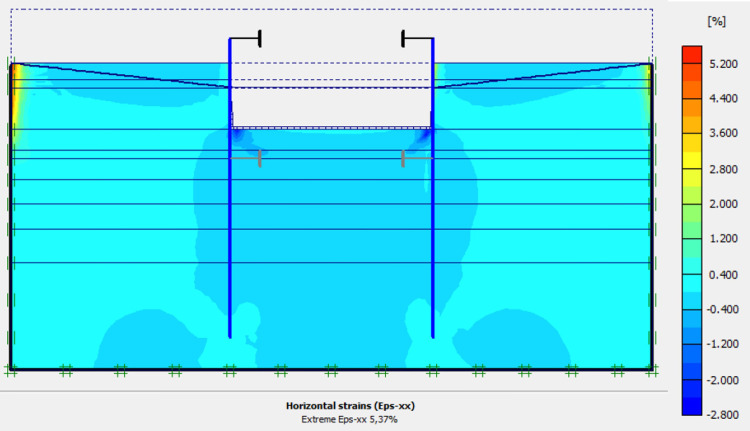
Shadings of the horizontal plastic strains of the model in phase 2.

This phase 2 is based on the fill with water of -4.0 to -1.5 m. In view of the results of this phase (Figs 13A–15), the variation of the quantities calculated compared to phase 1 remains small. The displacements and plastic strains of the model are almost identical to those of phase 1.

### 4.4. Phase 3: Excavation under water to– 7.0 m

**Fig 16:**
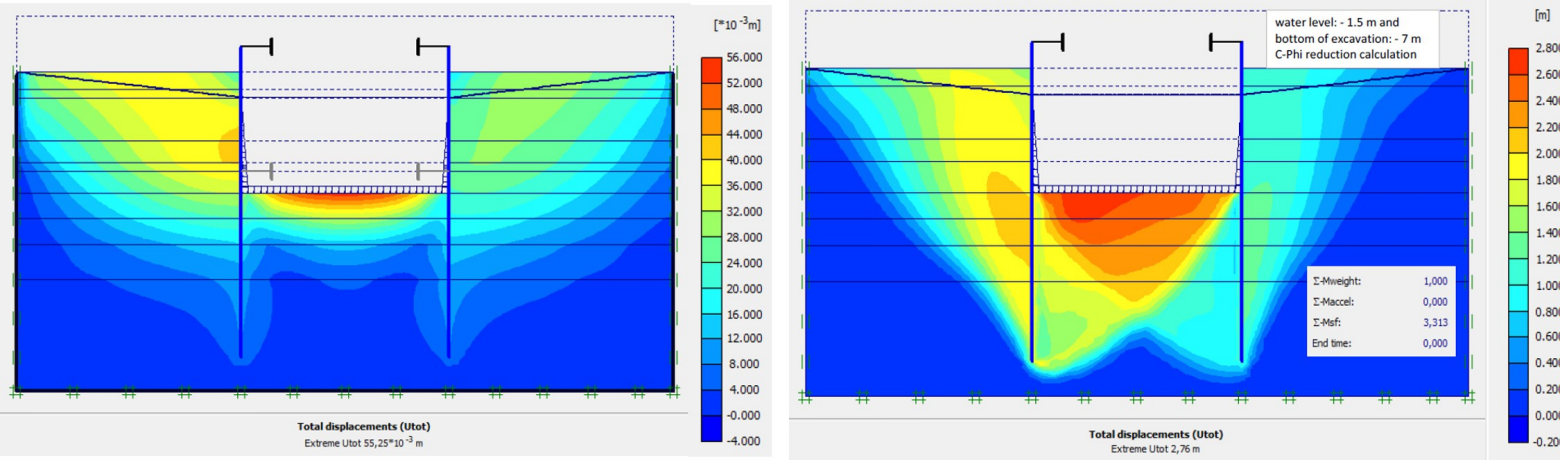
**a)** Shadings of the total displacements (m) of the model in phase 3; **b)** Shadings of the total displacements (m) of the model in phase 3 (c-phi reduction calculation—Factor of safety: 3.31).

**Fig 17:**
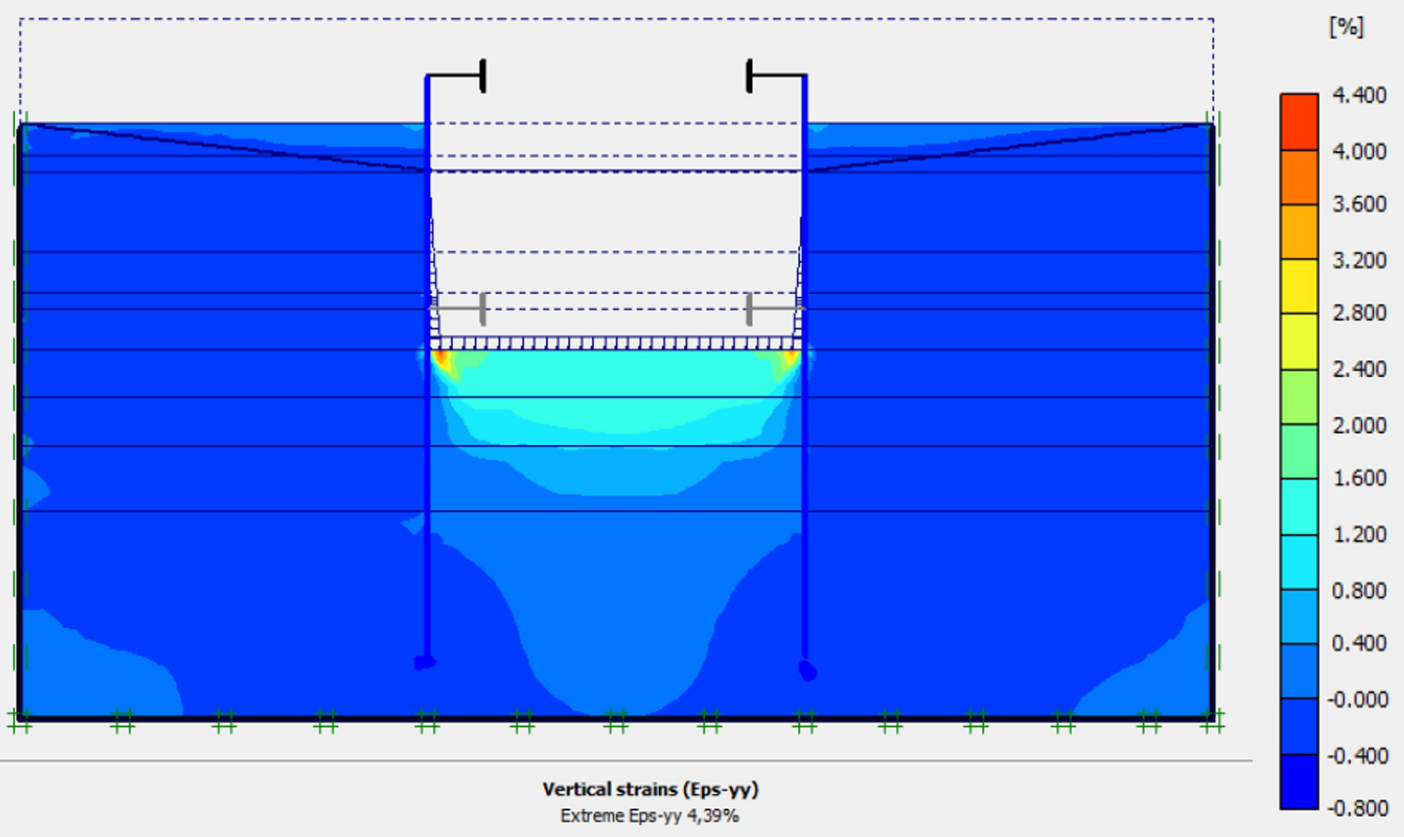
Shadings of the vertical plastic strains of the model in phase 3.

**Fig 18:**
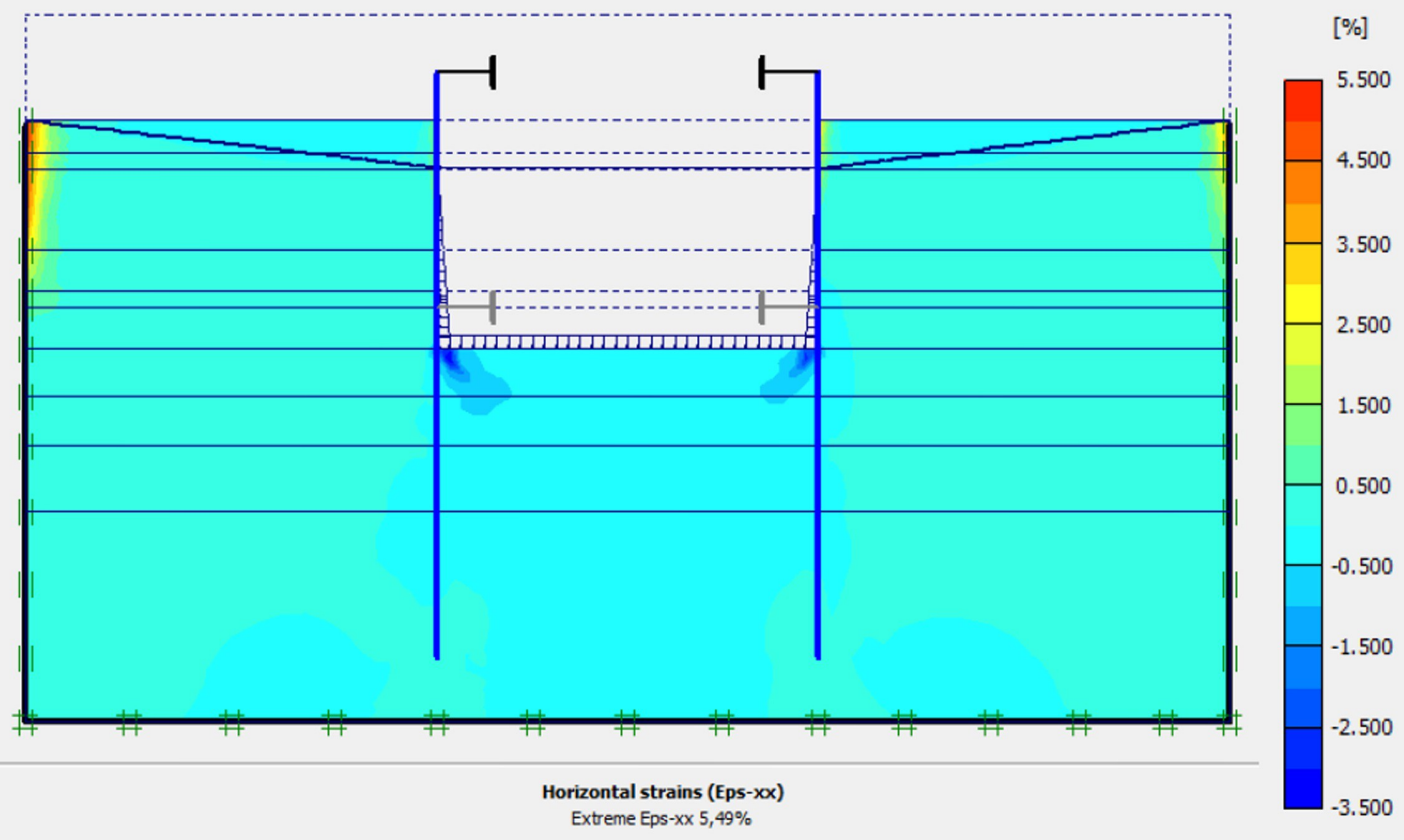
Shadings of the horizontal plastic strains of the model in phase 3.

**Fig 19:**
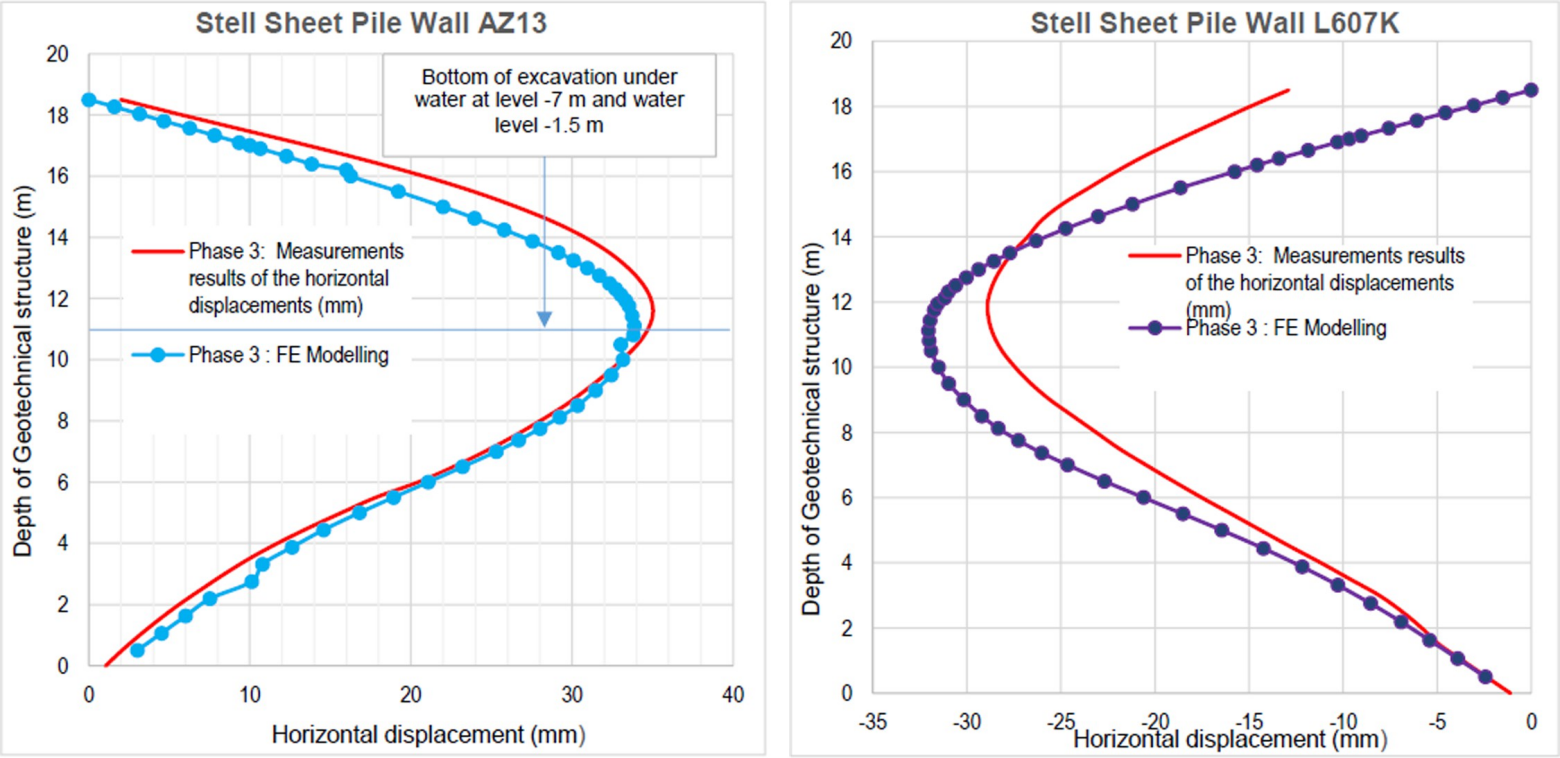
**a)** Horizontal displacement in the AZ13 stell sheet pile wall in phase 3; **b)** Horizontal displacement in the L607K stell sheet pile wall in phase 3.

This phase is based on the excavation under water of geostatic stress state in the ground. The excavation of the soft soils between the two walls induces a total displacement of 2.60 cm out of the excavation and about 5.44 cm inside the excavation. Figs 16A–18 present the total displacements and plastic strains (in phase 3) of the model. An increase in plastic strains around the walls is observed through Figs 17 and 18. The increase in deviatoric stresses as a function of the depth of the excavation also induces an increase in plastic strains around the excavated area. Perfect consistency is observed on horizontal displacements within the excavated area on the AZ13 walls (Fig 19). A slight difference is observed on the displacements of the L607K wall. The difference between the calculation and measurement results remains small. The horizontal displacements induced in this phase are significantly higher than those in phase 1.

### 4.5. Phase 4: Lowering water level to– 5.0 m

**Fig 20:**
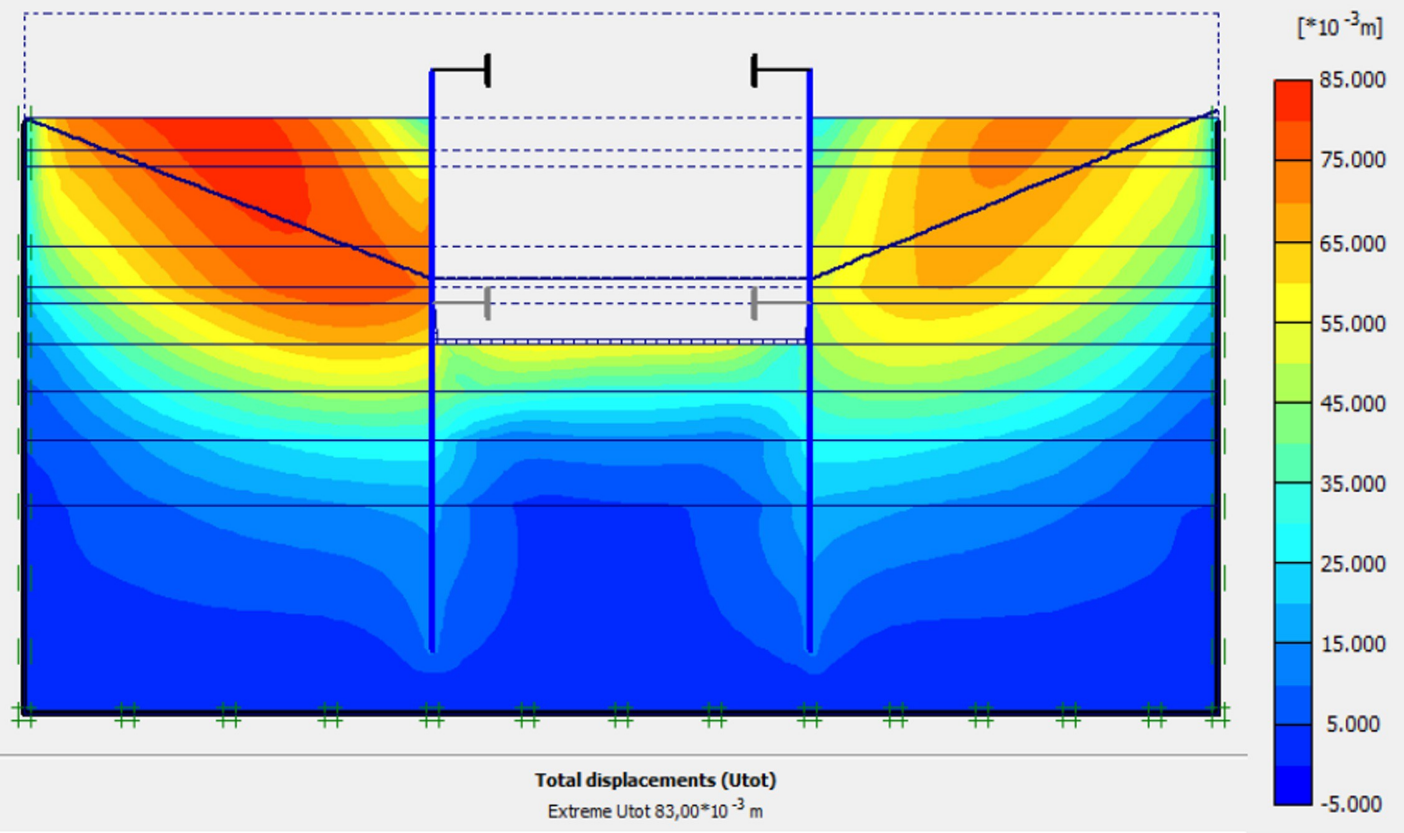
Shadings of the total displacements (m) of the model in phase 4.

**Fig 21:**
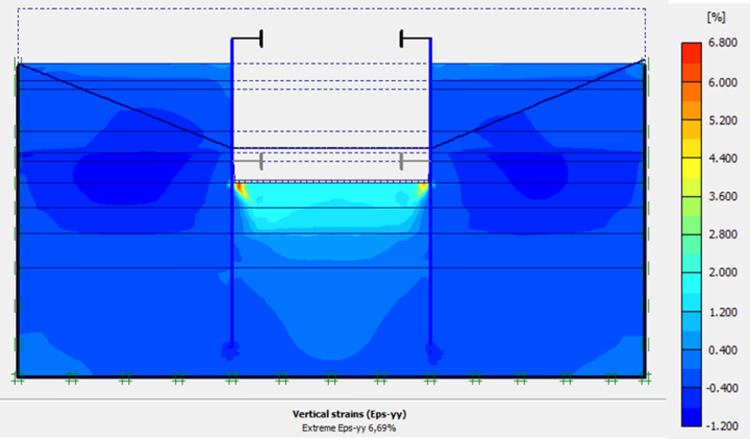
Shadings of the vertical plastic strains of the model in phase 4.

**Fig 22:**
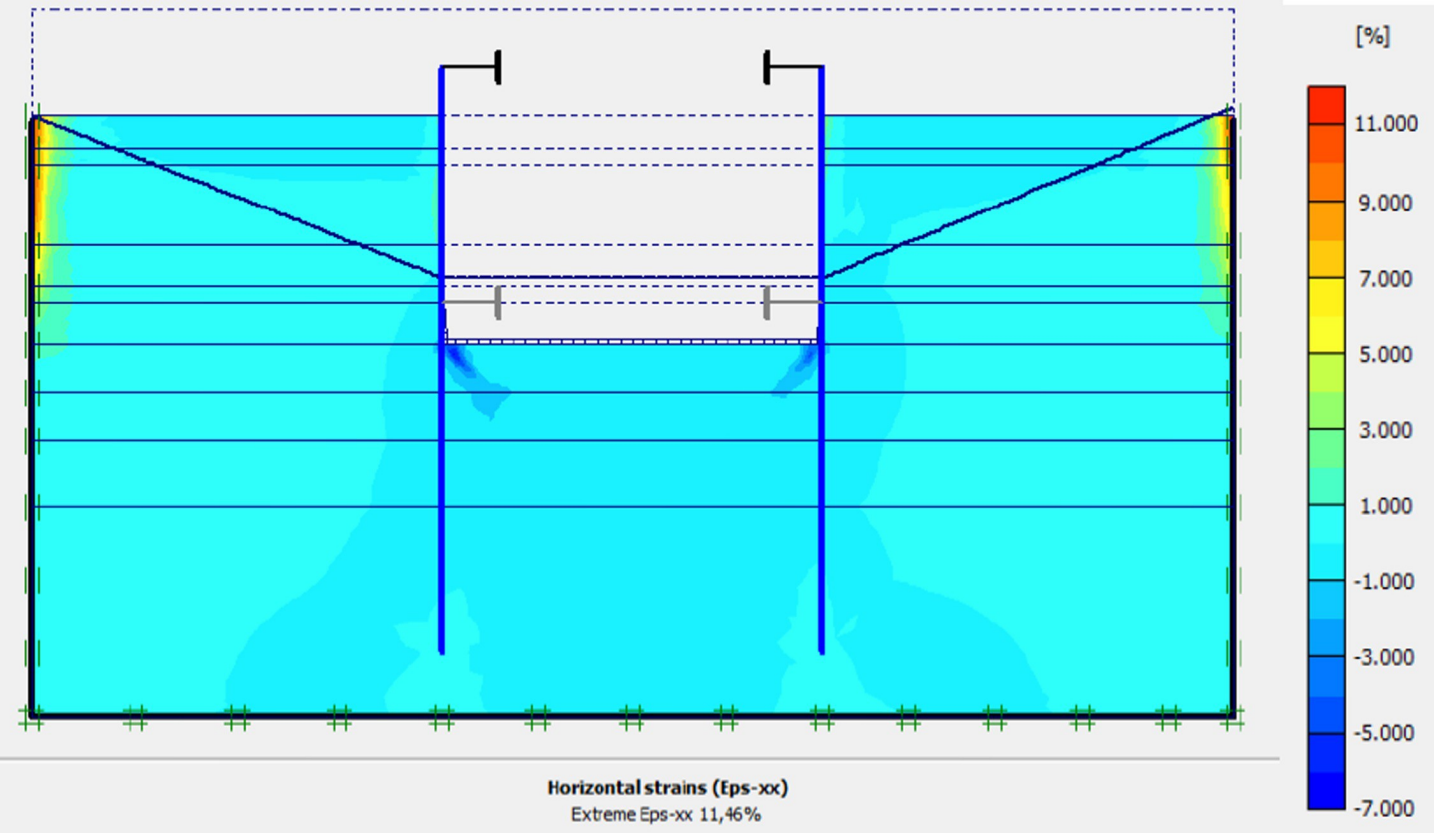
Shadings of the horizontal plastic strains of the model in phase 4.

**Fig 23:**
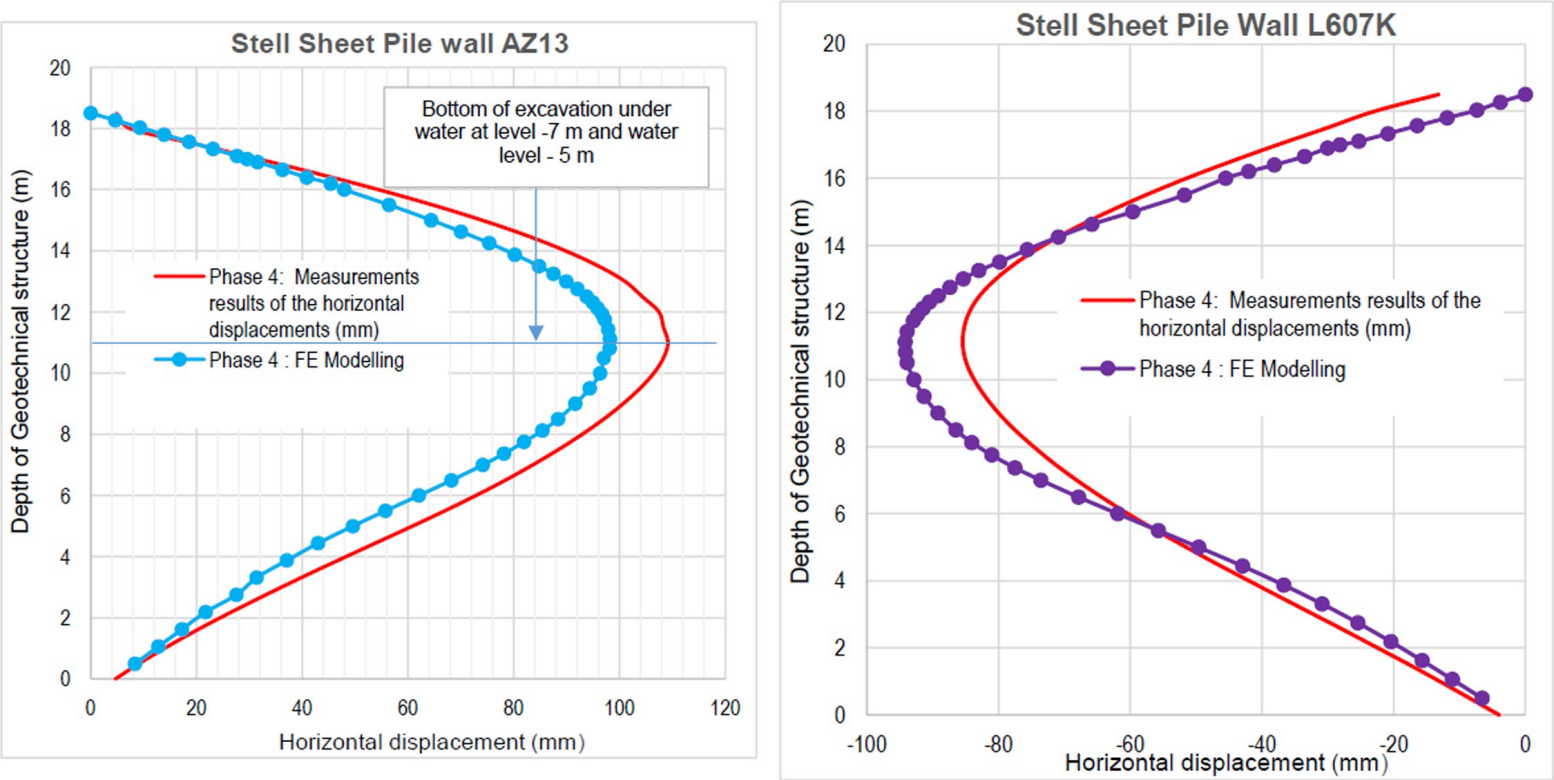
**a)** Horizontal displacement in the AZ13 stell sheet pile wall in phase 4; **b)** Horizontal displacement in the L607K stell sheet pile wall in phase 4.

In this phase 4, the lowering water of -1.5 to -5 m is realised. The drop in the water level from -1.5 to -5.0 m causes an increase in plastic stains, vertical and horizontal displacements within the excavation. The increase in horizontal displacements on the retaining walls is about 3 times compared to phase 3 (Figs 20–23). This unconfinement pressure causes strong instabilities around the retaining walls, despite the retention by struts at their tops. The values of plastic strains and lateral displacements on the walls are much higher than those expected for retaining structures (depending of the state-of-the-art).

### 4.6. Phase 5: Fill with water to -1.5 m

**Fig 24:**
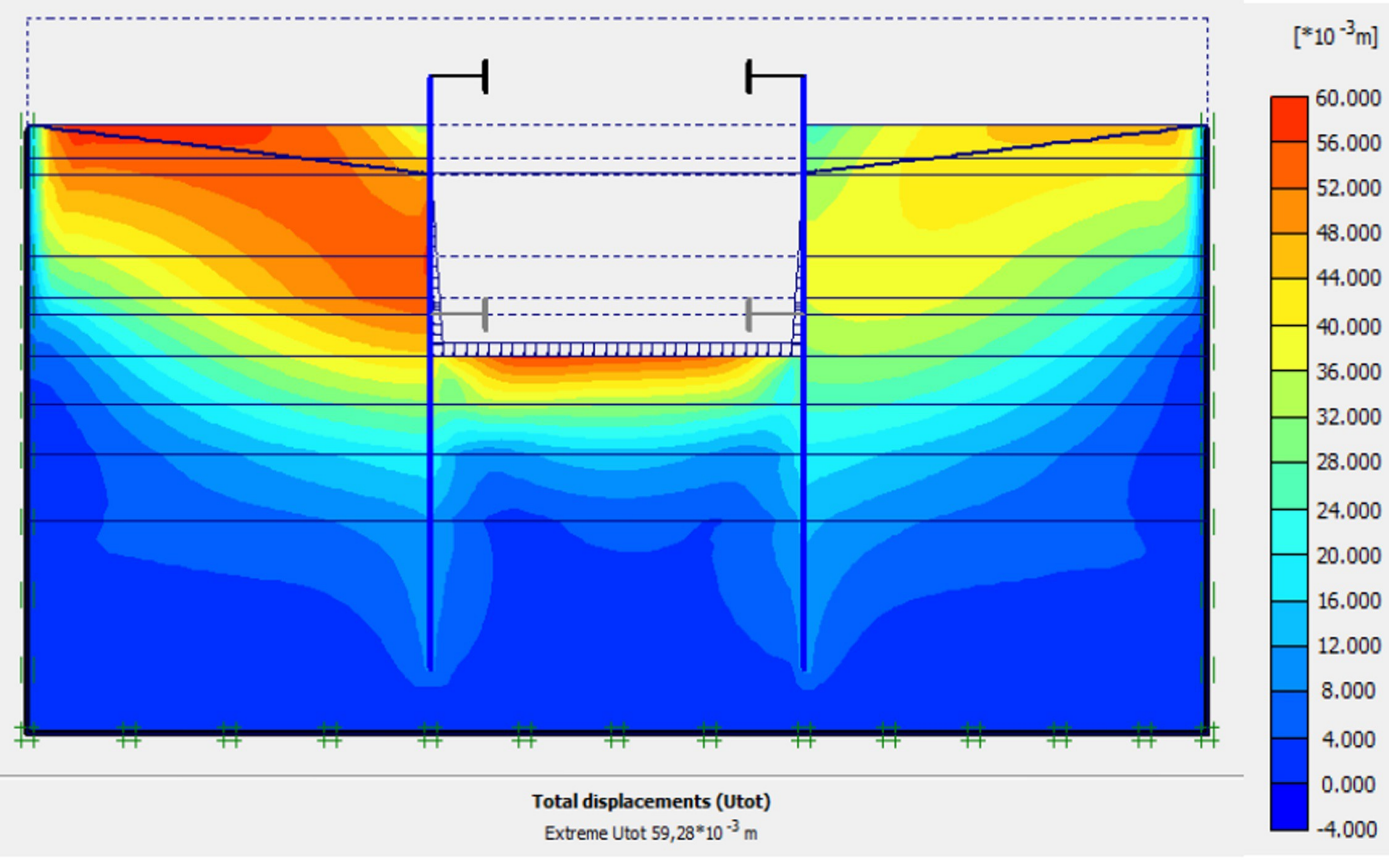
Shadings of the total displacements (m) of the model in phase 5.

**Fig 25:**
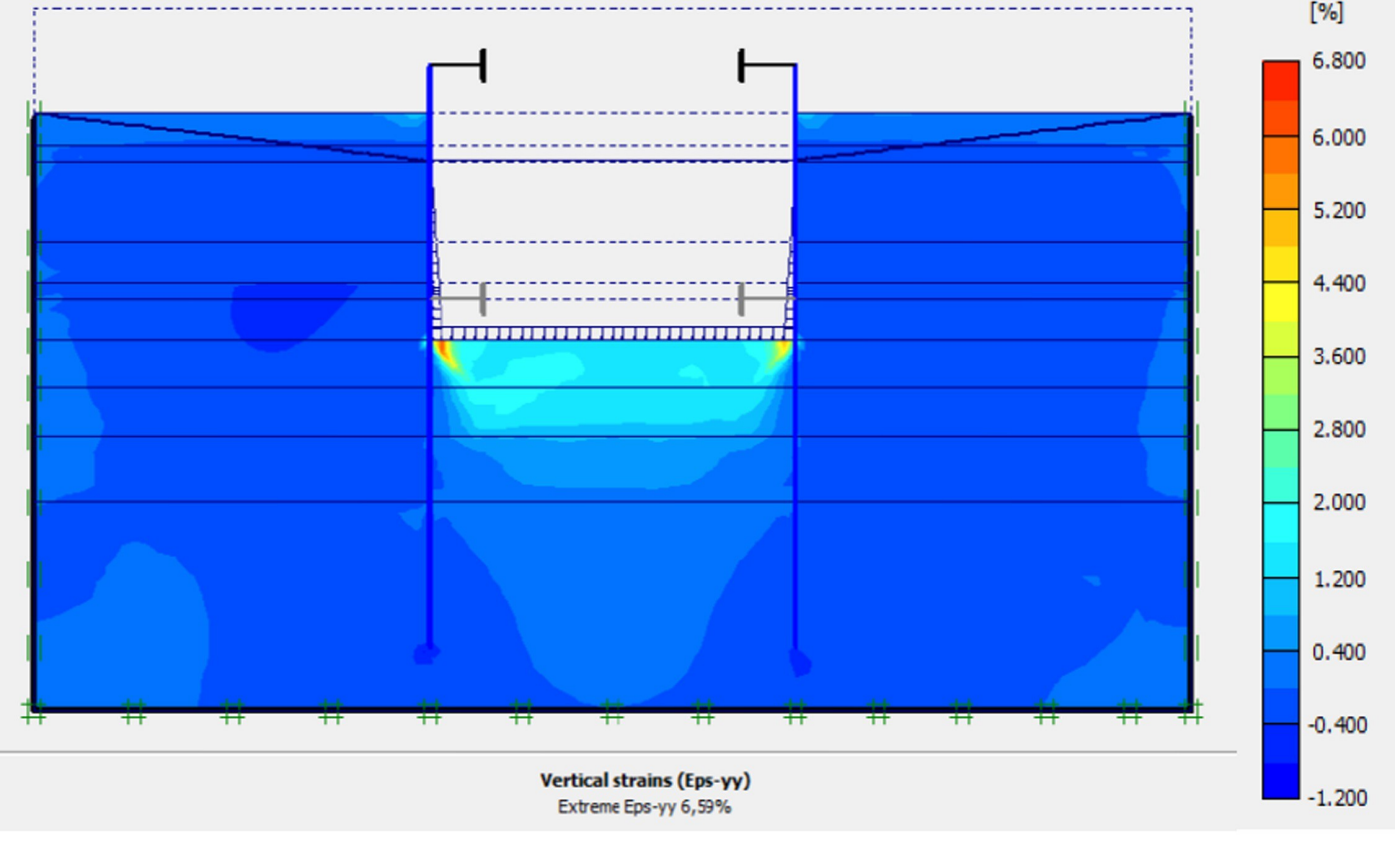
Shadings of the vertical plastic strains of the model in phase 5.

**Fig 26:**
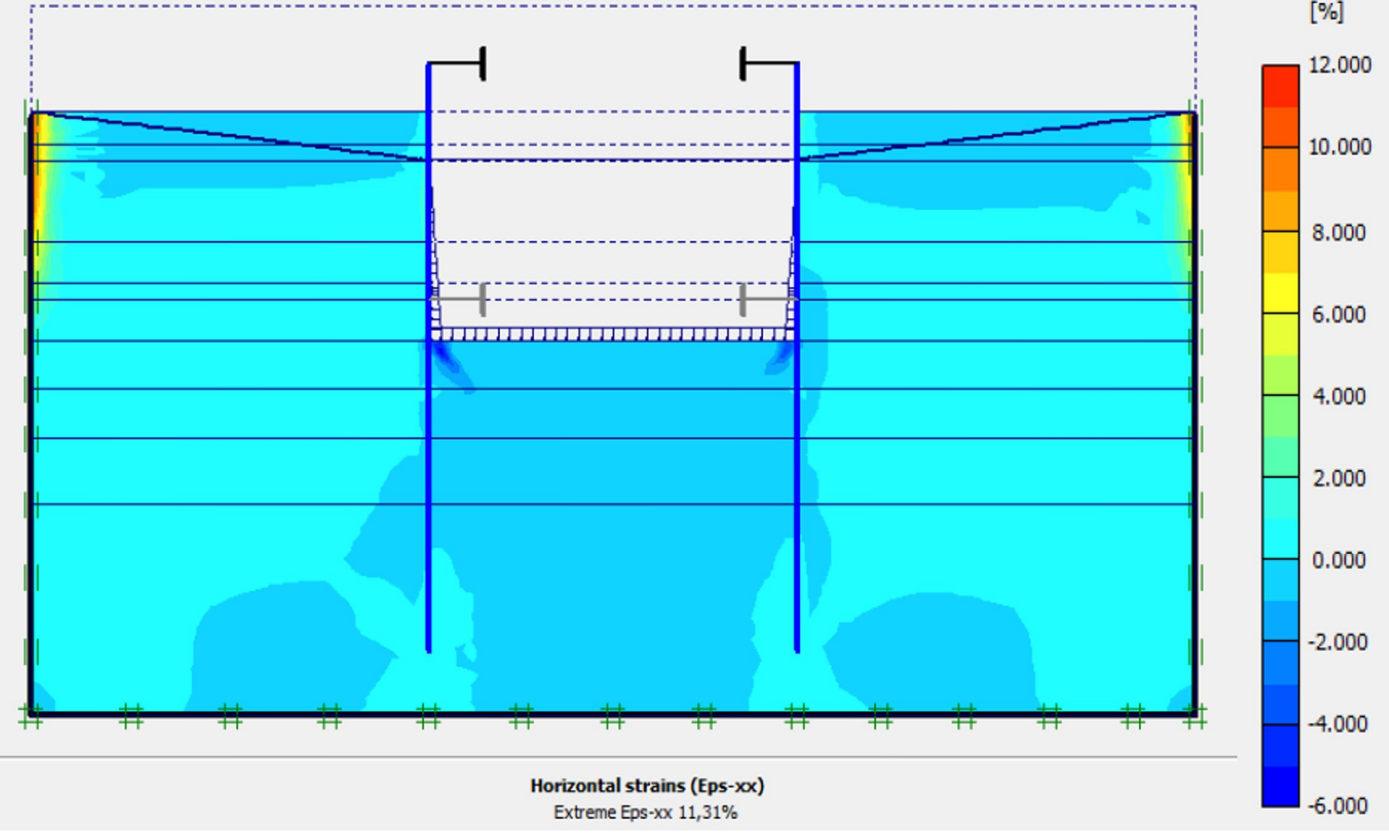
Shadings of the horizontal plastic strains of the model in phase 5.

The fill with water of -5.0 to -1.5 m is observed on the excavation in this phase 5. The variation of the quantities calculated (Figs 24–26) compared to phase 4 remains small. The displacements and strains of the model are almost identical to those of phase 4. The retaining walls having undergone excessive strains and horizontal displacements in phase 4, the fill with water operation from -5.0 to -1.5 m does not modify the apparent state of stresses in the soft soils.

### 4.7. Phase 6: Sand backfill behind AZ13 stell sheet pile wall

**Fig 27:**
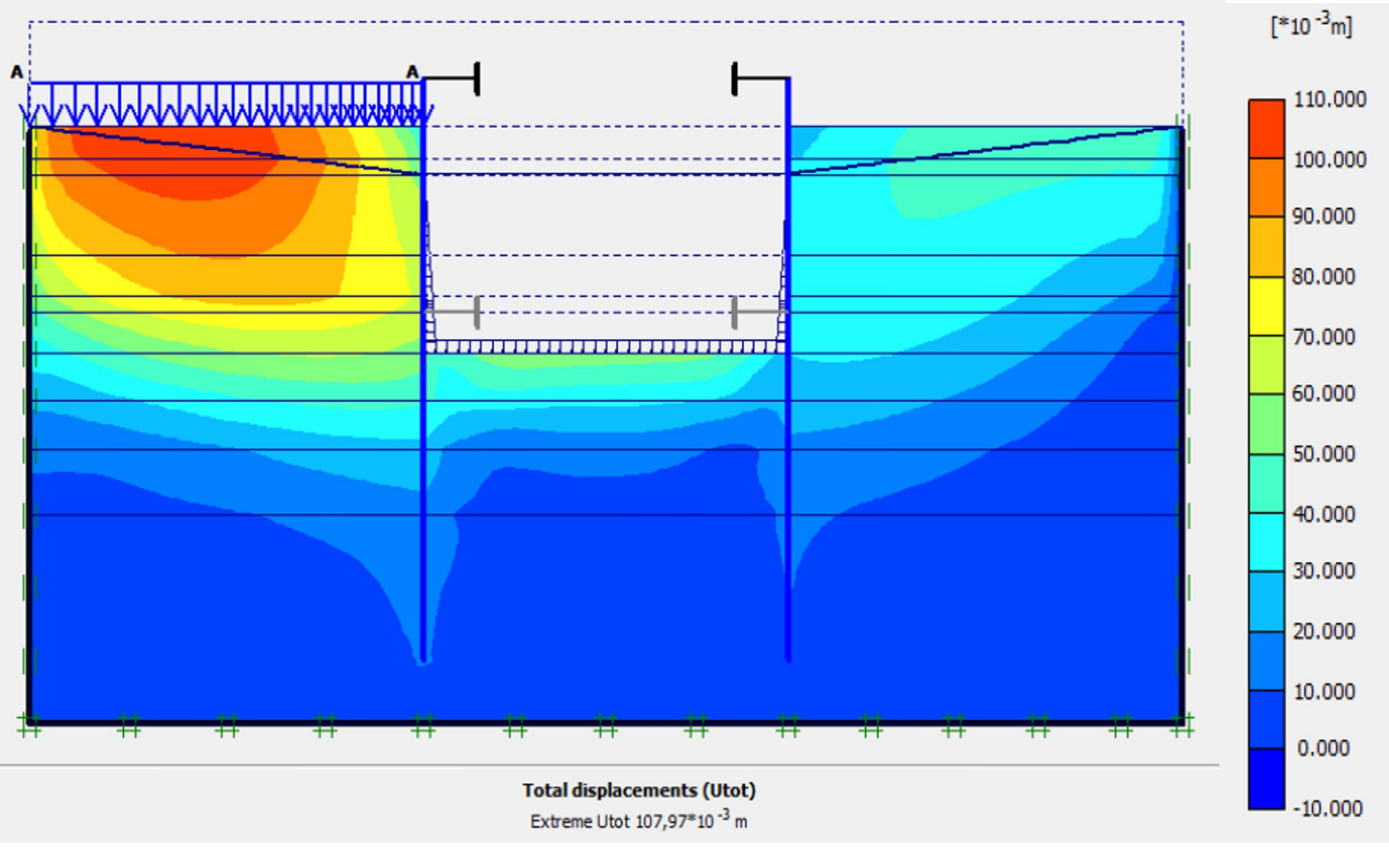
Shadings of the total displacements (m) of the model in phase 6.

**Fig 28:**
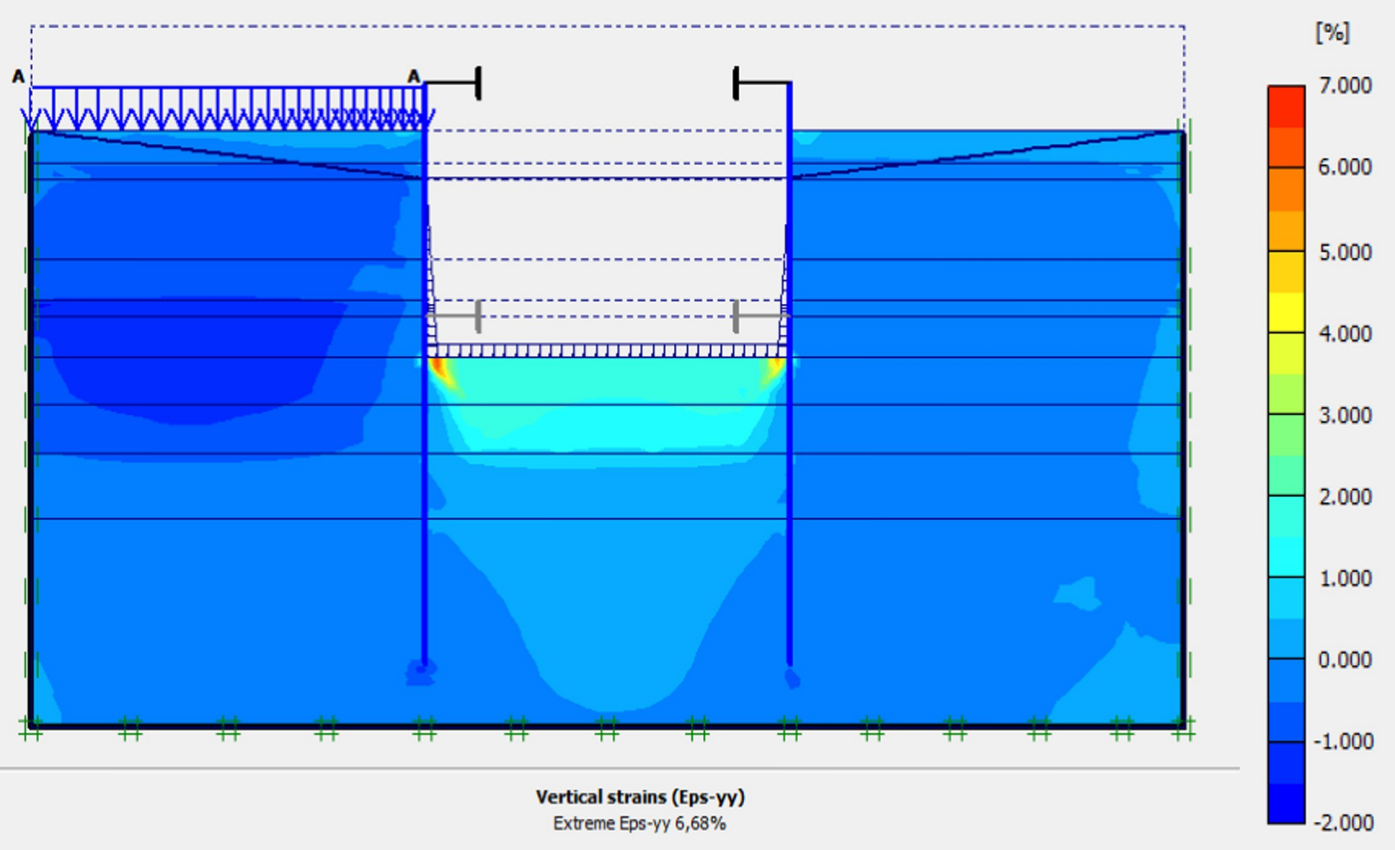
Shadings of the vertical plastic strains in phase 6.

**Fig 29:**
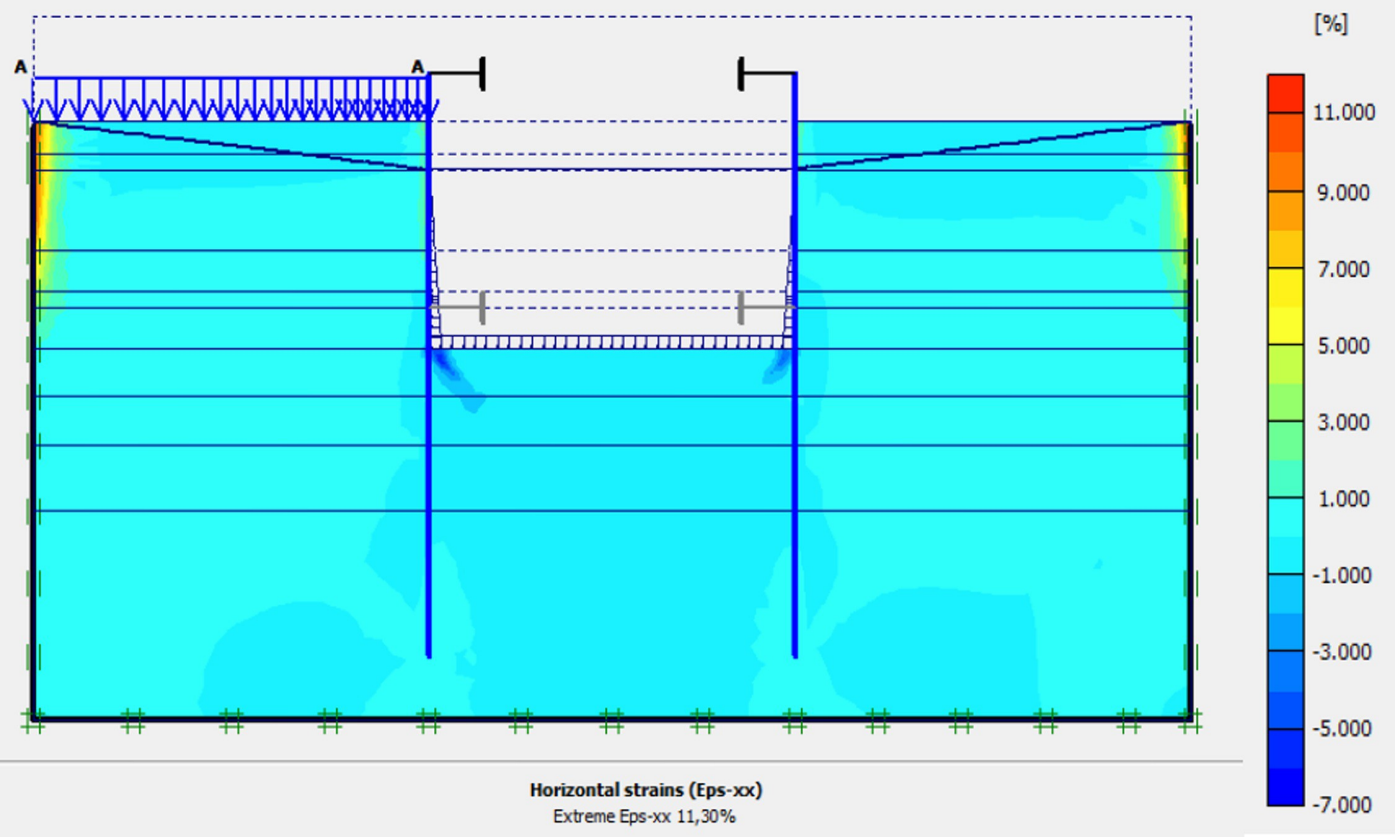
Shadings of the horizontal plastic strains of the model in phase 6.

**Fig 30:**
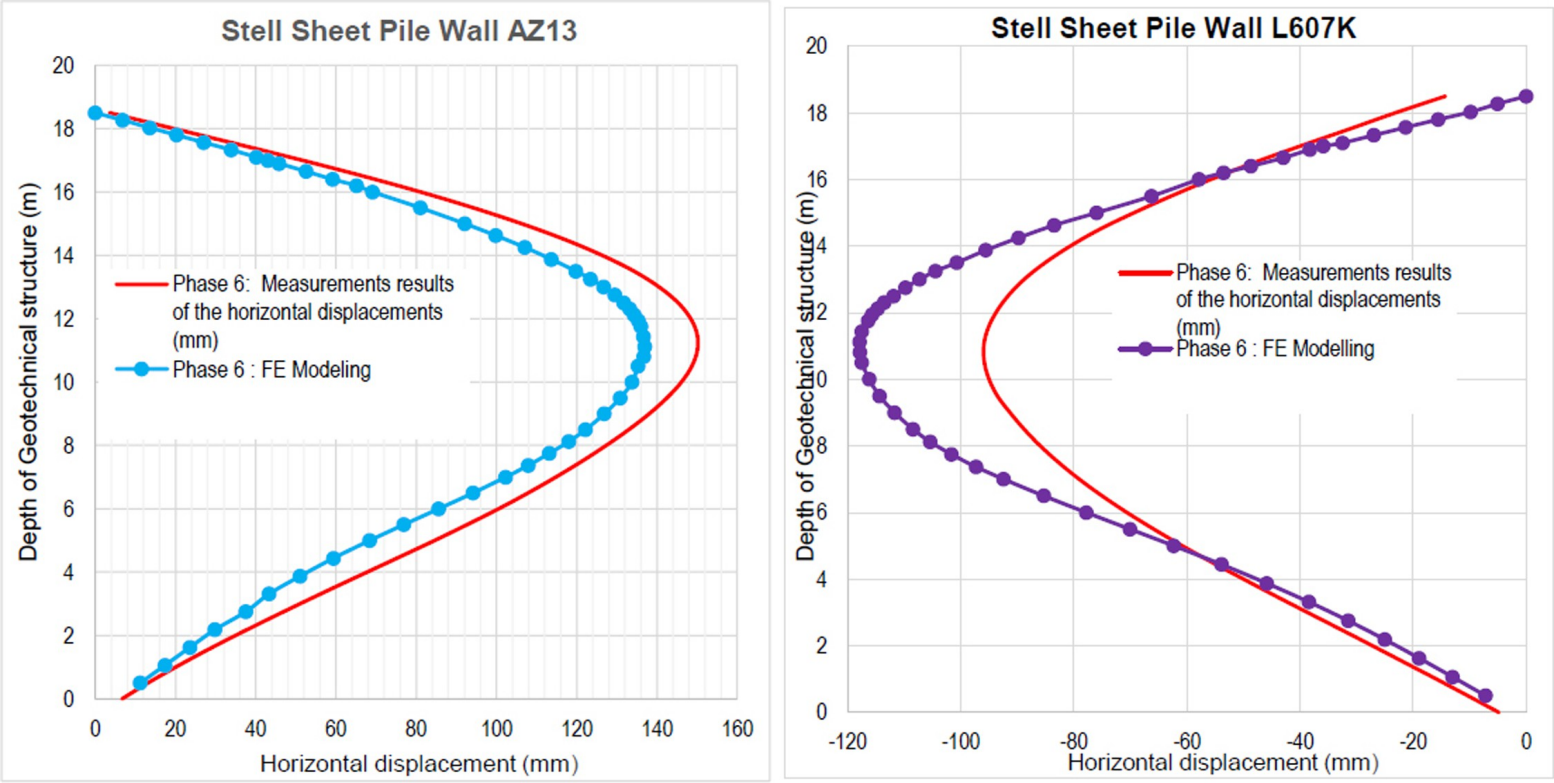
**a)** Horizontal displacement in the AZ13 stell sheet pile wall in phase 6**; b)** Horizontal displacement in the L607K stell sheet pile wall in phase 6.

In this phase 6, the application of surface load for sand backfill behind AZ13 stell sheet pile wall is realized. Figs 27–30 present the total displacements, plastic strains and horizontal displacements in the walls. The total displacement behind the AZ13 wall reaches 10.8 cm. It remains of the order of 4.5 cm behind the L607K wall. This sand backfill creates a great increase in stresses, strains and displacements around the AZ13 wall. An increase of about 39% in horizontal displacements on the AZ13 wall is observed compared to phase 4 (Fig 30).

### 4.8. Phase 7: Lowering water level to– 5.0 m

**Fig 31:**
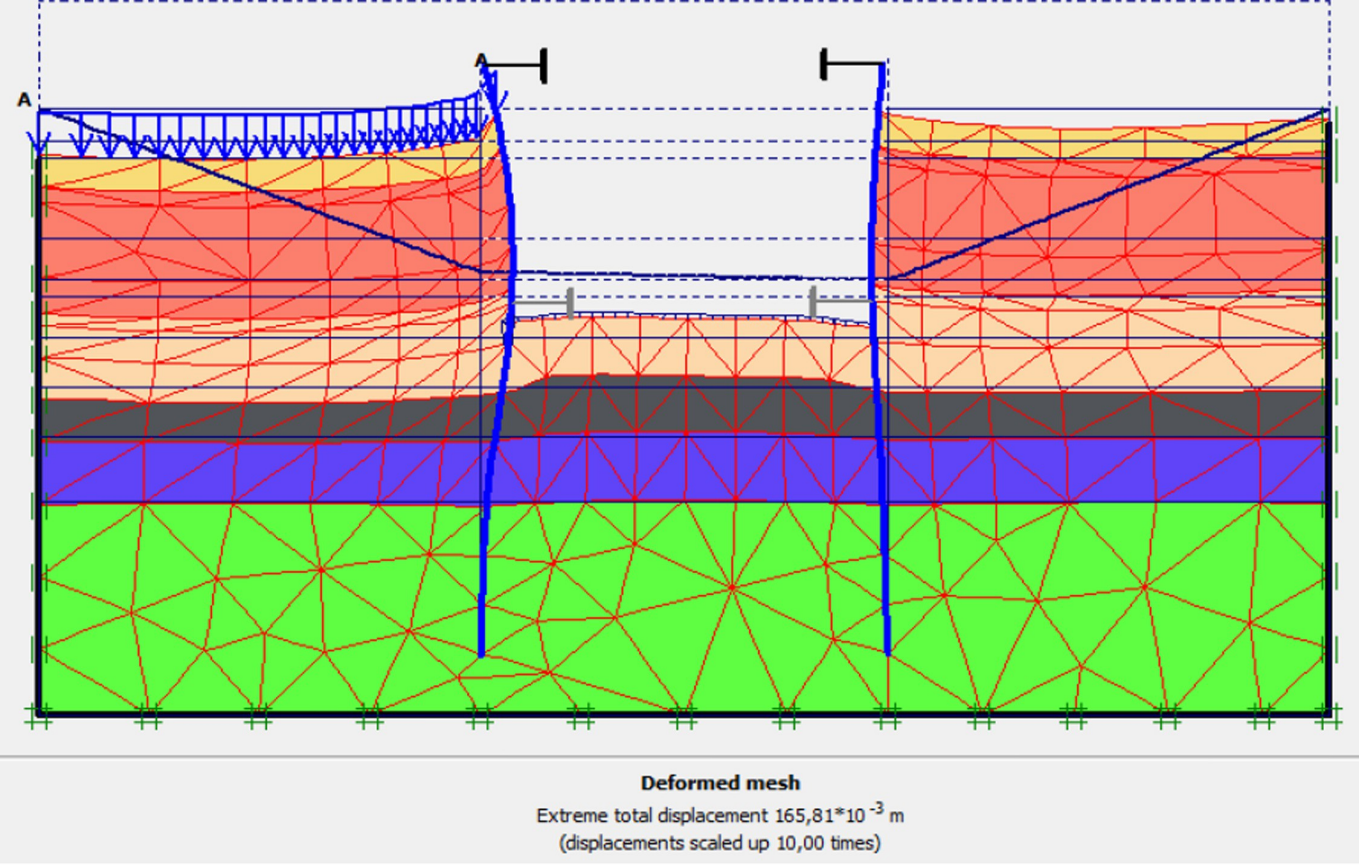
Deformed mesh of the model in phase 7.

**Fig 32:**
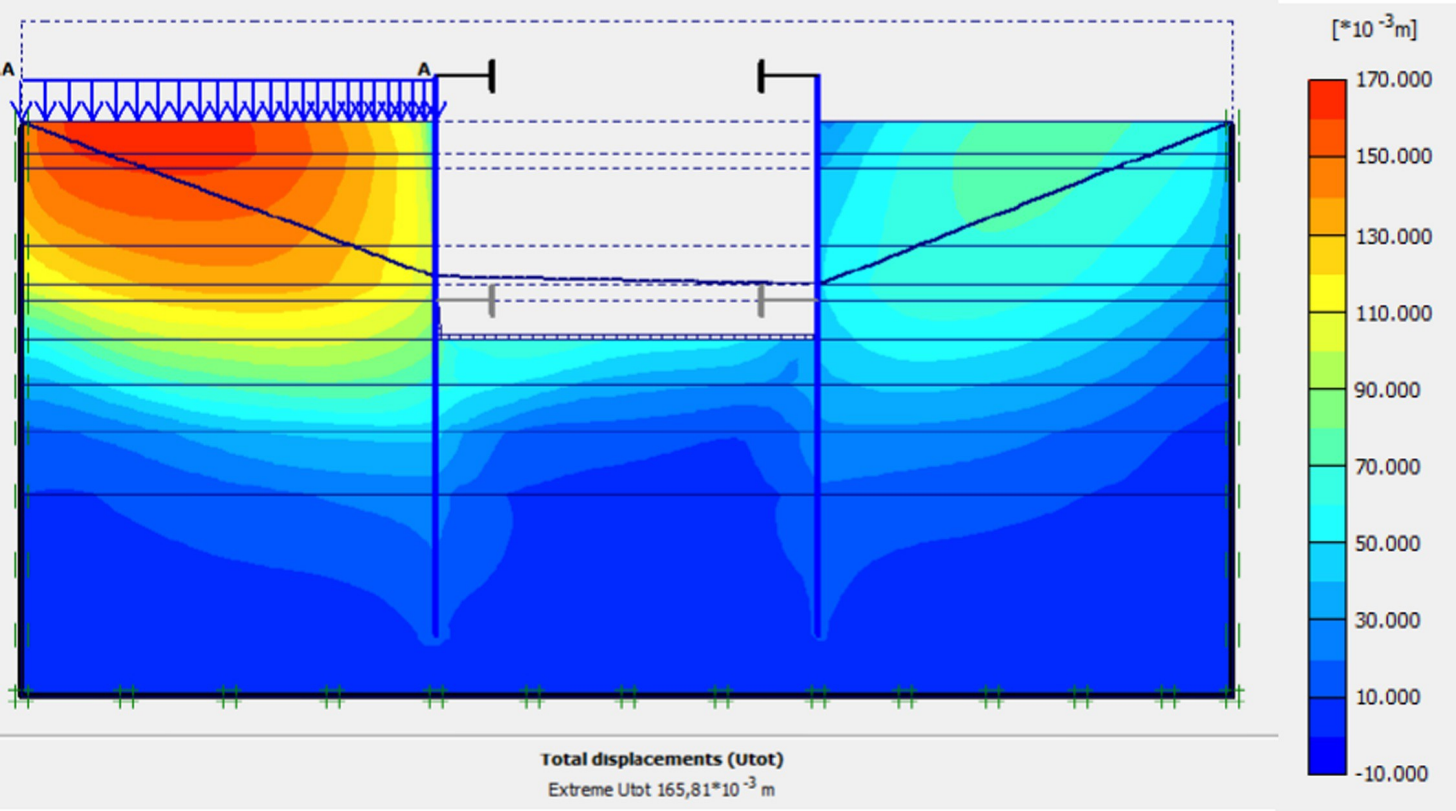
Shadings of the total displacements (m) of the model in phase 7.

**Fig 33:**
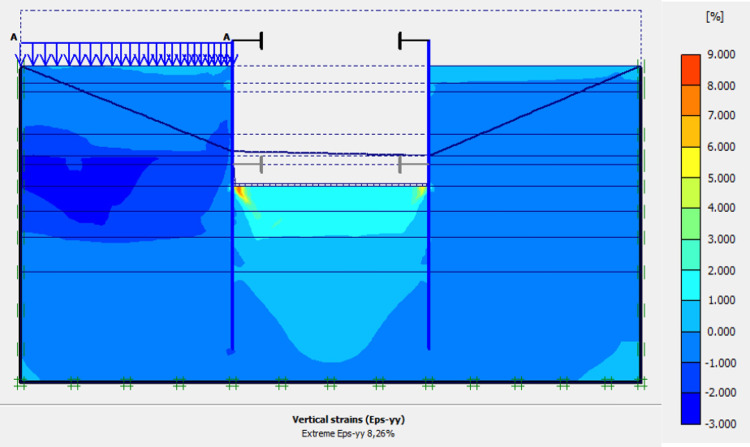
Shadings of the vertical plastic strains in phase 7.

**Fig 34:**
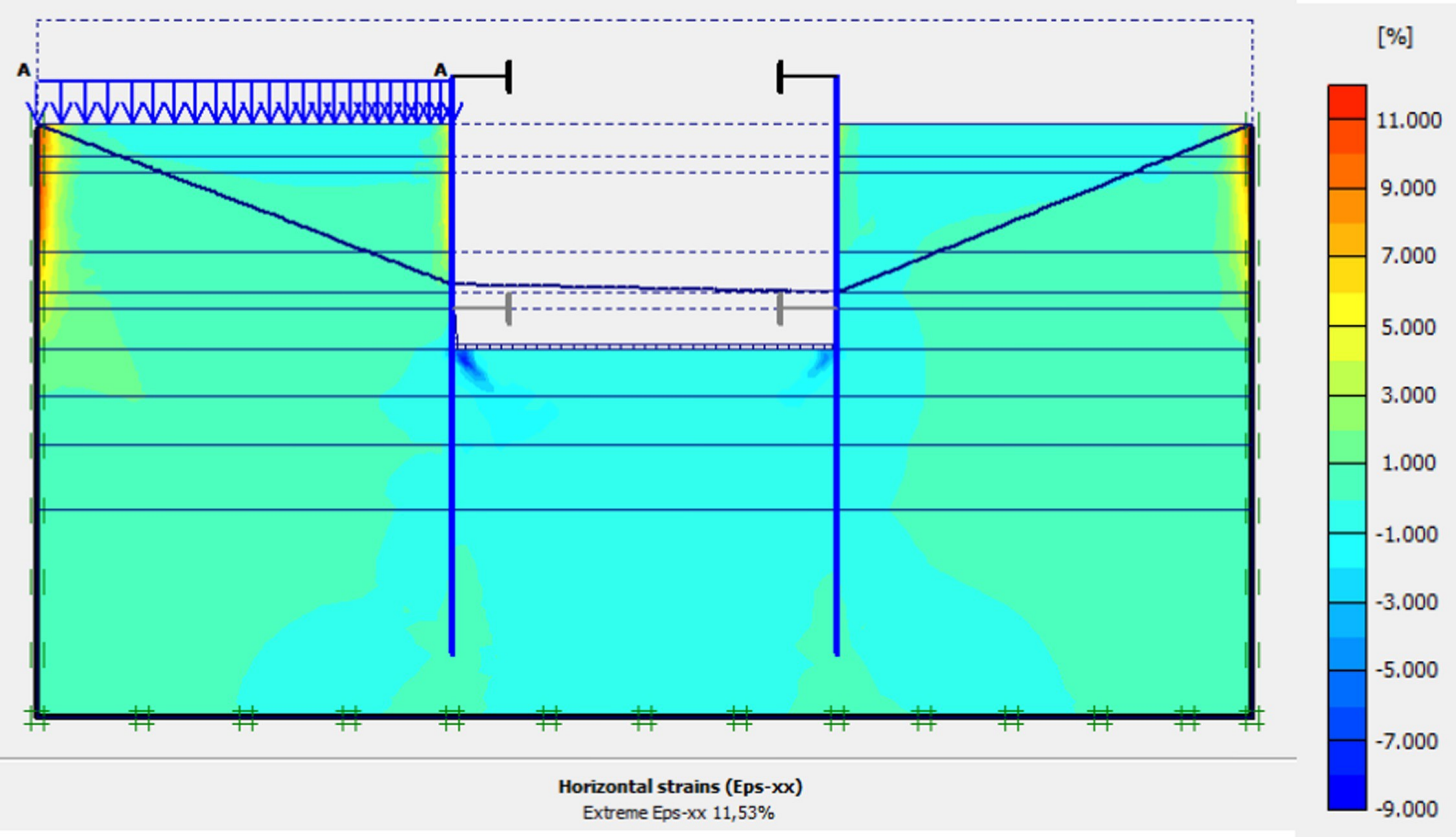
Shadings of the horizontal plastic strains of the model in phase 7.

The lowering water of -1.5 to -5 m is realised. Figs 31–34 presents the deformed mesh, the total displacements and plastic strains of the model. The drop in water level from -1.5 to -5.0 m causes an increase in total displacements (16.58 cm), plastic strains around the AZ13 wall. A slight increase in these sizes is observed around the L607K wall as well.

### 4.9. Phase 8: Long term performance–Bottom of excavation and water level at -7 m

**Fig 35:**
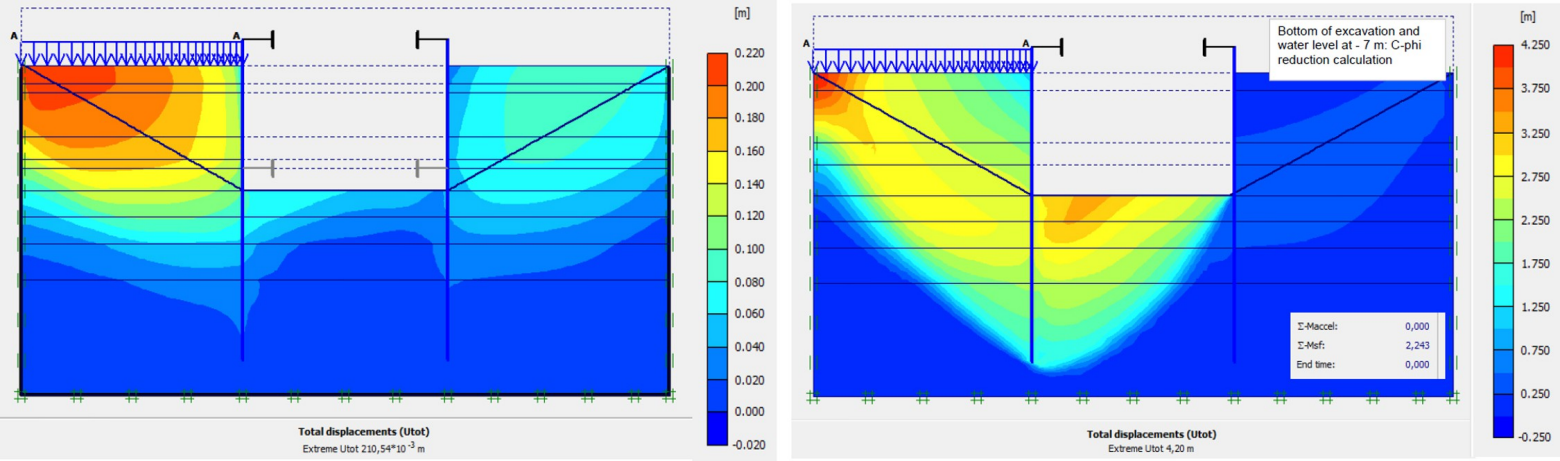
**a)** Shadings of the total displacements (m) of the model in phase 8; **b)** Shadings of the total displacements (m) of the model in phase 8 (c-phi reduction calculation—Factor of safety: 2.24).

**Fig 36:**
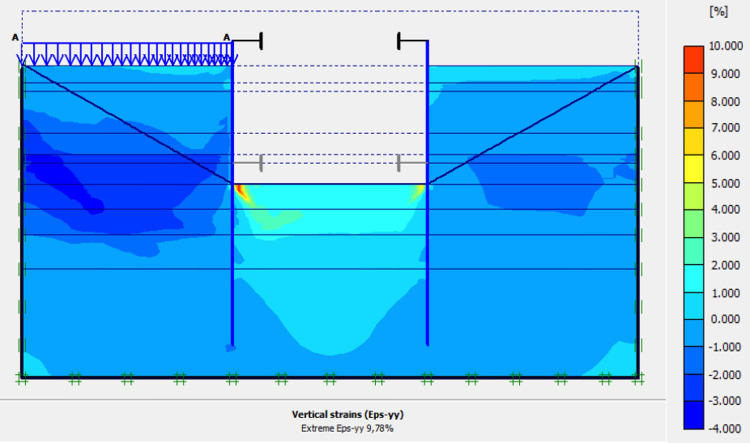
Shadings of the vertical plastic strains in phase 8.

**Fig 37:**
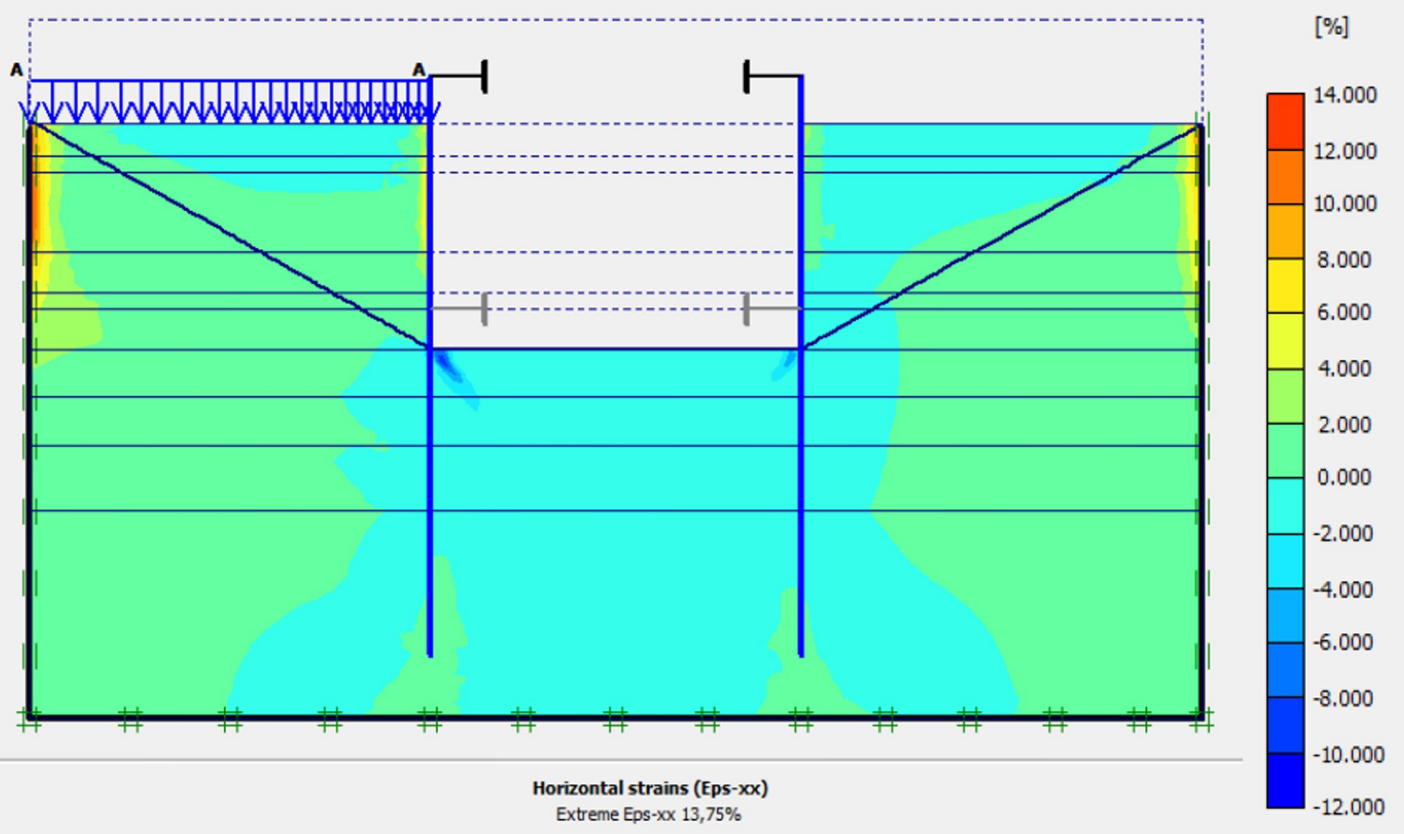
Shadings of the horizontal plastic strains of the model in phase 8.

**Fig 38:**
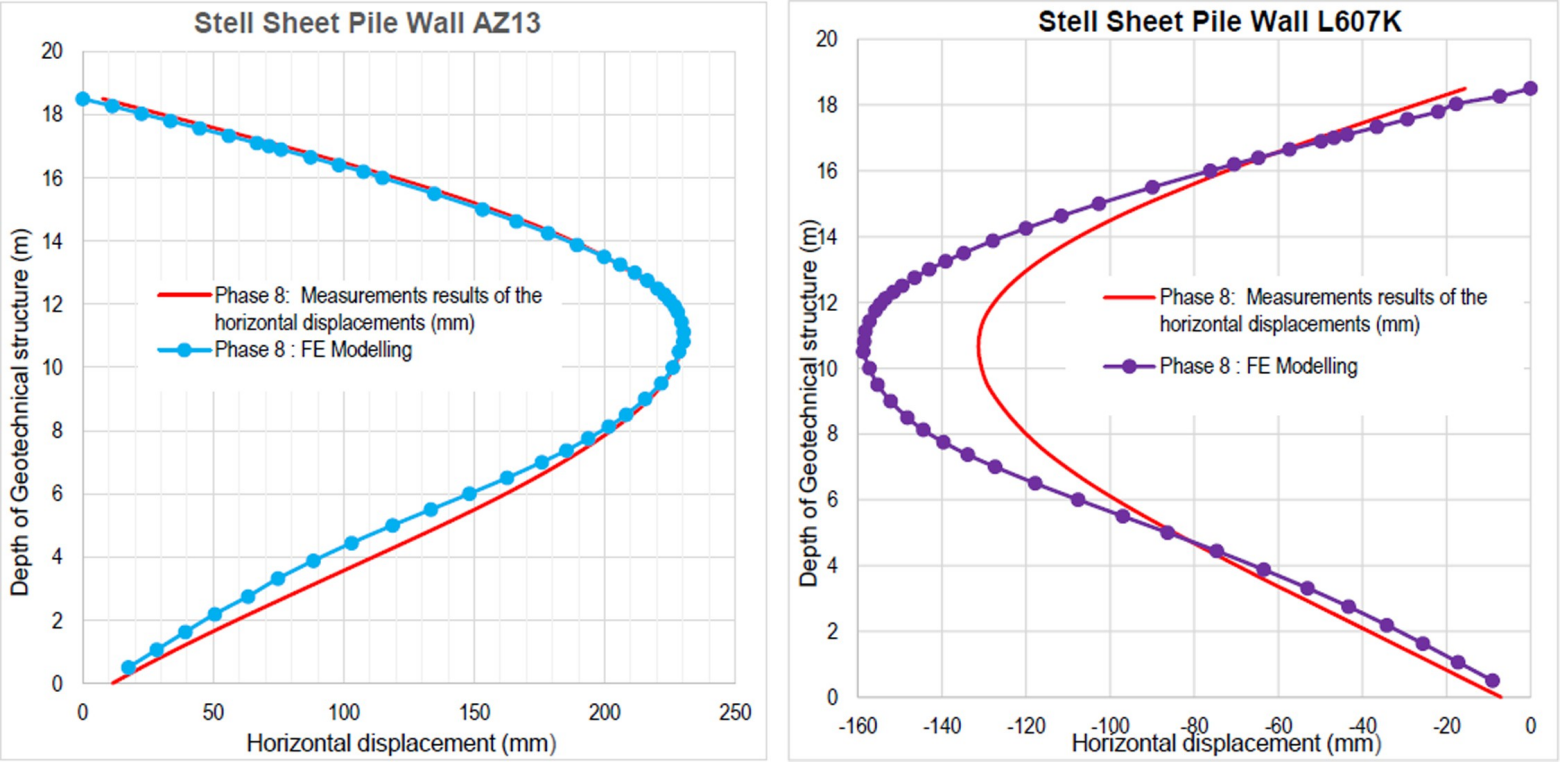
**a)** Horizontal displacement in the AZ13 stell sheet pile wall in phase; **b)** Horizontal displacement in the L607K stell sheet pile wall in phase 8.

**Fig 39:**
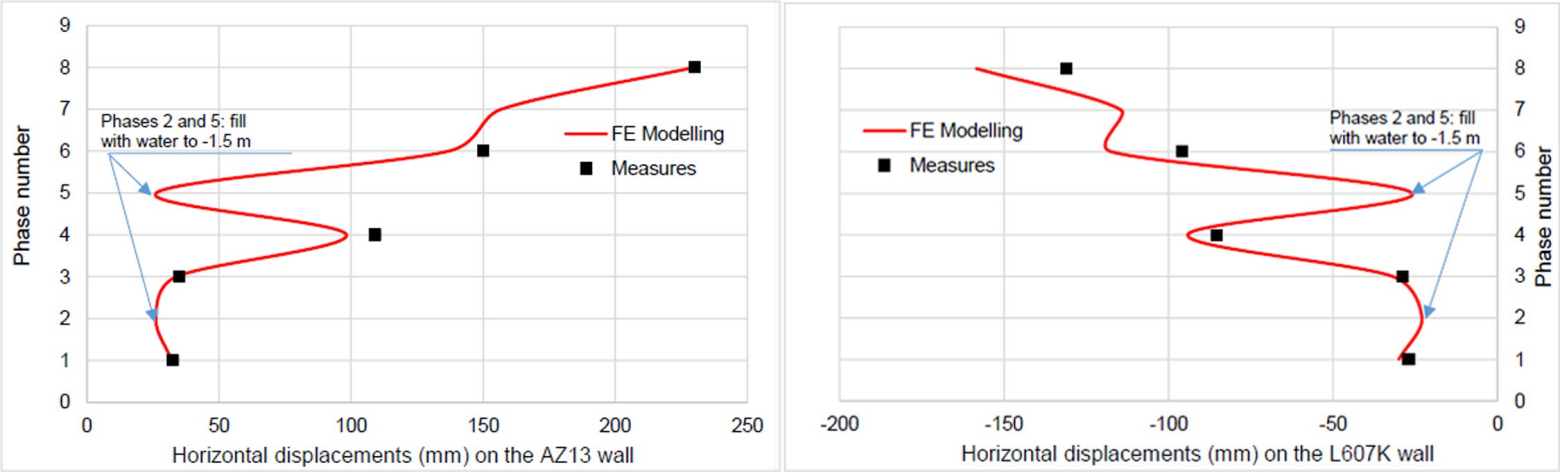
**a)** Horizontal displacements in the AZ13 stell sheet pile wall in phases 1.2 to 8; **b)** Horizontal displacements in the L607K stell sheet pile wall in phases 1.2 to 8.

The calculation of the long-term behavior of the structure is performed with the gradual lowering of the water level to the bottom of the excavation (-7.0 m). This drop also causes an increase in total displacements (21.01 cm behind AZ13 stell sheet pile), plastic strains, and large horizontal displacements on the retaining walls (Figs 35A–38B). These horizontal displacements are of the order of 230 mm on the AZ13 wall (against 137 mm in phase 6) and 159 mm for the L607K wall (against 118 mm in phase 6) (Figs 38A and 39).

### 4.10. Phase 9: Blocking of horizontal displacements of the walls at—5.75 m depth from the ground surface in the excavation

**Fig 40:**
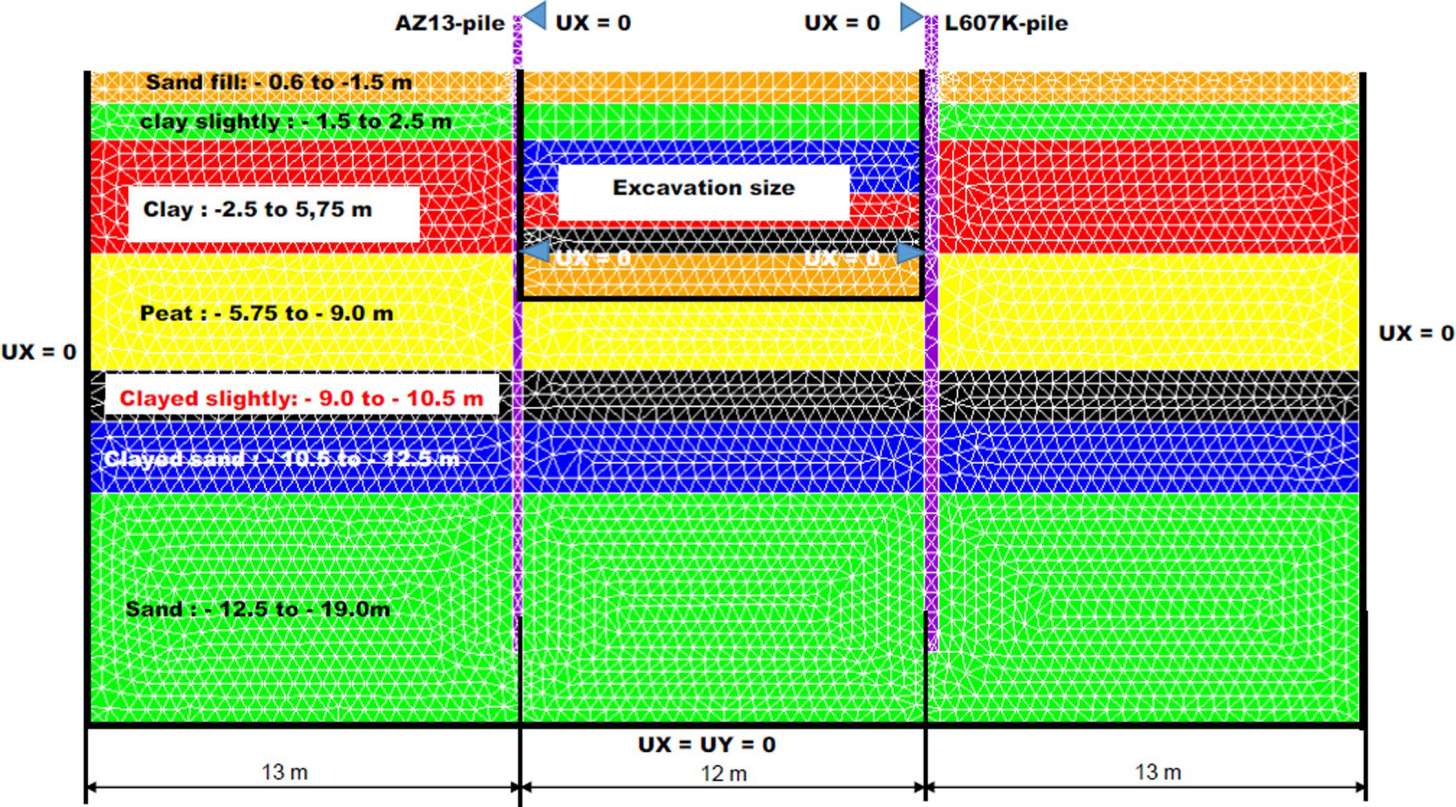
Detailed view of the 2D mesh and soil clusters of the deep excavation in phase 9 (fixiting by struts at a level 5.75 m).

**Fig 41:**
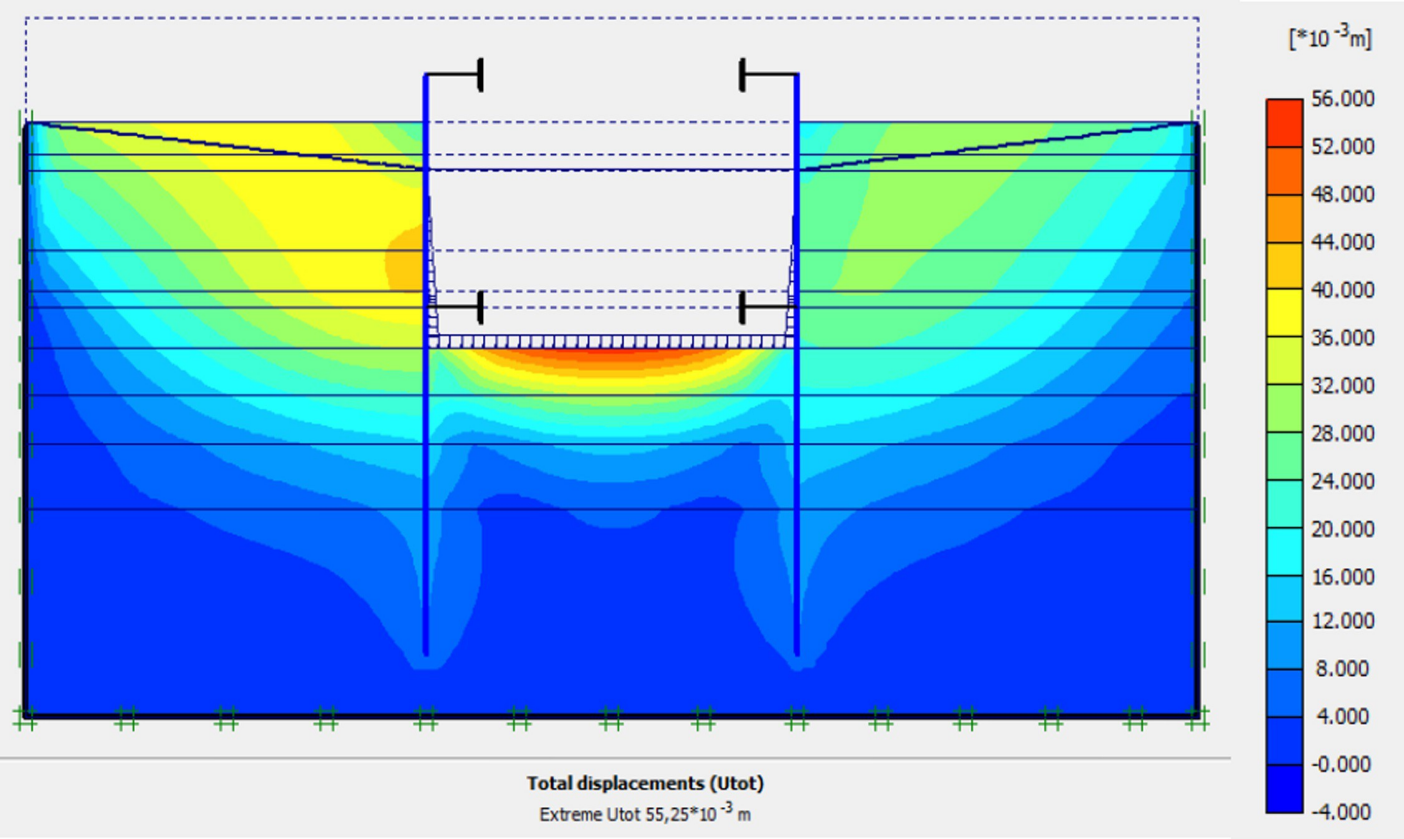
Shadings of the total displacements (m) of the model in phase 9.

**Fig 42:**
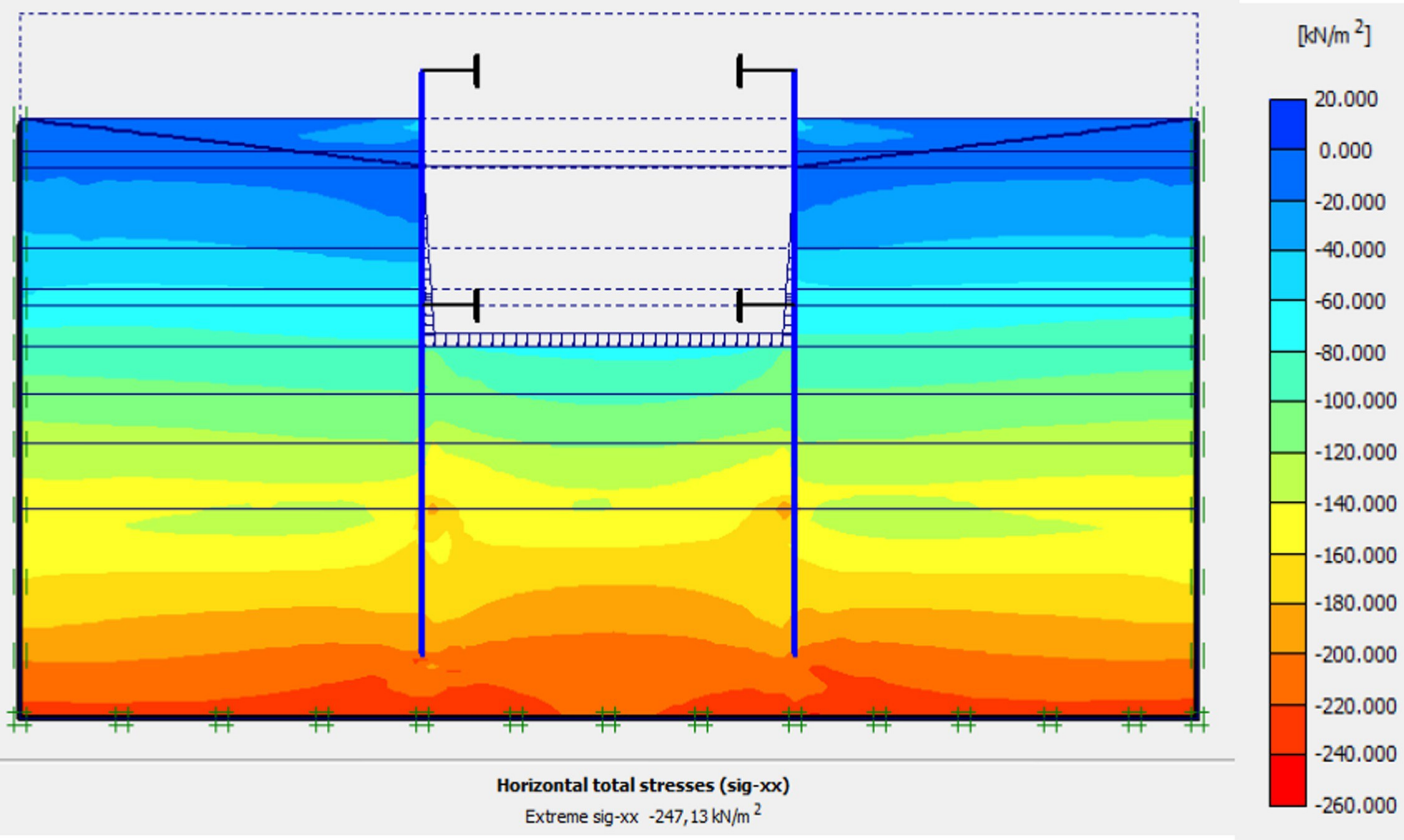
Shadings of the horizontal stresses of the model in phase 9.

**Fig 43:**
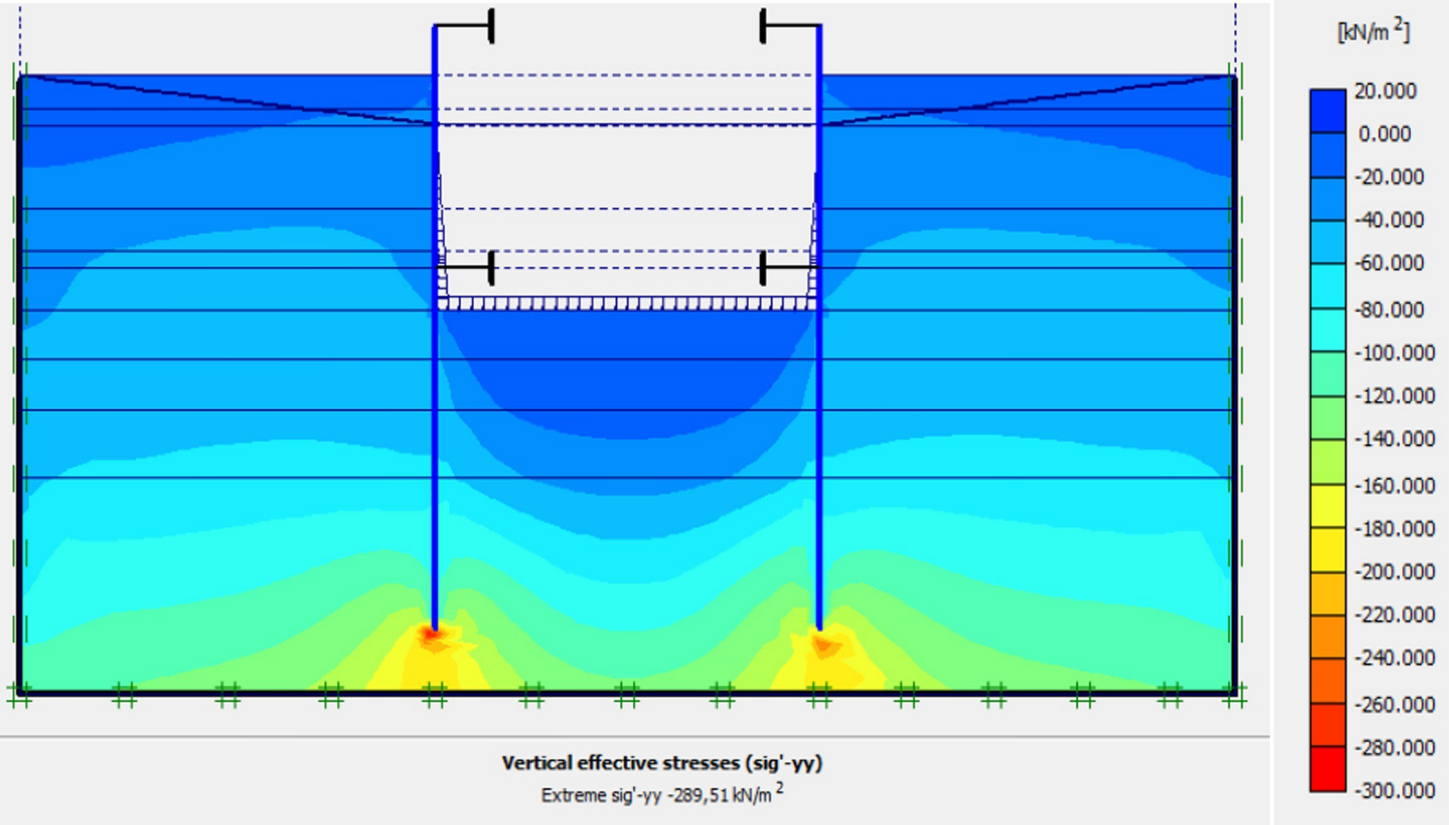
Shadings of the vertical stresses of the model in phase 9.

Figs 40–43, display the detailed view of the 2D mesh of the deep excavation in phase 9 (fixiting by struts at a level 5.75 m), the deformed mesh, horizontal and vertical stresses of the model.

## 5. Discussion

In this study, it can be noted that the values of horizontal strains obtained on the stell sheet piles walls greatly exceed (see Table 4) those commonly accepted for such structures which are of the order of 10^−4^ to 10^−3^ [76]; hence the need for another struts fixing these large strains. The horizontal displacement curves of the test wall were determined in the site from inclinometer measurements [18]. All significant values regarding the state-of-the-art requirements are presented in Table 4 and Figs 5–43. They can be compared by those obtained by various authors around the world (Table 5) and the formulas 6 to 15. The figures show that the maximum values of horizontal strains and horizontal displacements are located in the peat layer whose machanical properties are poor (Figs 7, 11, 12, 15, 18, 19, 22, 23, 26, 29, 30, 34 and 37–39) or at the bottom of excavation. The finite element and measurements of the displacements for AZ13 and L607K walls are illustrated in Figs 12, 19, 23, 30, 38 and 39. The results of the horizontal displacements for all phases on the AZ13 and L607K walls are illustrated on the Fig 39. For phases 2 and 5 (water rise to– 1.5 m), the horizontal displacements were not measured. The differences between the FE calculation and measurements results for the horizontal displacements water pressures and lateral earth pressure are small.

**Table 4:** Results of FE calculations on deep excavation retained by stell sheet pile walls.

Phases description	Phase	Stell sheet pile wall AZ13				Stell sheet pile wall L607K				s (mm) bottom of excavation	v (mm) behind AZ13	v (mm) behind L607K wall
		ux (mm)	ux max /H	εxx (%)	σx (kPa)	ux (mm)	ux max /H	εxx (%)	σx (kPa)			
Dry excavation to– 4.0 m (free fixiting in the walls)	1.1	35.43	0.0071	-	452	45.24	0.0091	-	292.9	-	**-**	**-**
Dry excavation to– 4.0 m	1.2	32.09	0.0064	3.00	766.6	-30	0.006	2.50	623.5	54.5	**-48.22**	**-52.34**
Fill with water to -1.5 m	2	24.26	0.0052	2.80	562.7	-23	0.0046	2.00	472.5	**53.80**	-22.46	-23.24
Excavation under water to– 7.0 m	3	33.86	0.0042	3.50	651.5	-32	0.004	3.00	532.7	**54.42**	-25.95	-25.28.
Lowering water level to– 5.0 m	4	98.24	0.0123	7.00	1021	-94	0.0118	5.00	792.7	56.57	**-62.40**	-57.47
Fill with water to -1.5 m	5	25.24	0.0033	6.00	586.7	-26	0.0033	4.00	497.3	57.22	**-28.63**	-26.02
Sand backfill behind AZ13 wall	6	136.98	0.0171	7.00	893.6	-117	0.0147	5.00	462.2	56.46	**-** **96.03**	-25.86
Lowering water level to– 5.0 m	7	156.94	0.0196	9.00	1433	-144	0.0143	6.50	740.8	61.97	**-155.76**	-61.81
Long-term (water level to -7 m)	8	**230.18**	**0.0288**	**12.0**	**1708**	**-158**	**0.0198**	**8.00**	**862.1**	**62.20**	**-202.51**	**-79.70**
Fixiting by a 2^nd^ strut at -5.75 m	9	20.9	0.0026	4.00	1188	-30	0.0038	4.00	571	53.99	-26.11	-25.30

The explanations provided for Table 4 are: Horizontal tensile stresses (σ_x_) in the walls, settlement or lifting of the bottom of excavation (s), horizontal strains (ε_x_), horizontal displacements (u_x_), vertical displacement behind walls (v). The values of $\frac{V_{\max}}{H}$ varies between 3.26 x 10^−3^ to 2.53 x 10^−2^ behind AZ13 and 3.16 x 10^−3^ to 1.05 x 10^−2^ behind L607K and $\frac{s_{\max}}{H}$ vary between 6.75 x 10^−3^ to 1.09 x 10^−2^ at the bottom of excavation.

**Table 5:** Horizontal displacements (u_x_) and displacement behind walls (v) in the literature.

Author	Soil type	location	ux max/H	v max/H
Zoa and Amba [17]	Lateritic clay	Cameroon	0.003	-
Moorman [35]	sands	-	0.0025	-
Moorman [36]	Stiff clays	-	0.005	-
Marten [37]	Various soils	France	< 0.0025	-
Clough and O’Rourke [38]	Various soils	FE analysis	< 0.002	0.001
Finno and Harahap [39]	Various soils	Chicago, USA	0.014	0.021
Ou et al., [40–32]	Various soils	Taipei	0.002–0.005	0.005–0.007
Carder [43]	Hardening soil	London, GB	0.0013–0.004	**0.001–0.002**
Wong et al., [44]	Soft soils	Singapore	0.005	0.0035
Nejjar [48]	Soft soils	Paris, France	0.0007	-
Burlon [49]	Various soils	Paris, France	-	0.5 to 2 u_max_
El Arja [50]	Various soils	Paris, France	0.0003	0.0007
Schweiger [51]	Sand	Berlin, Germany	0.001	0.0005–0.0015
Peck [54]	Soft soils	-	0.02	0.02
Mestat et al., [65]	All soils	Serviceability Limit State	0.002	-

The earth and water pressures did not increase substantially as a result of the sand backfill. The extra load caused a pressure increase on the test wall but due to the large strain capacity of both the soft soil ground and the wall itself, the extra pressure was immediately transferred into wall displacement and active earth pressure condition was obtained once again. The direct response of the construction to refilling of the excavation is observed. The displacement in the wall gave an immediate and large response as the excavation was refilled. This is mainly due to the large strain capacity of the soft ground together with undrained soil response. The shadings of the vertical plastic strains (Figs 6, 10, 14, 17, 21, 25, 28, 33 and 36) shows that excessive plastic strains are localized in the peat layer which has very poor geotechnical characteristics and the bottom of excavation. This behavior is justified by the fact that the stell sheet pile walls are blocked at their apex inside the excavation and the first horizontal stresses appear on the first parts in contact with the soil ground. The middle of the so-called stell sheet pile walls becomes flexible depending on the depth of the excavation. The results of on-site measurements through the horizontal displacements curves on the L607K stell sheet pile wall show a loosening of the struts which causes the appearance of horizontal displacements at the apex to the excavated side.

The results of the numerical horizontal displacements through the curves presented show zero displacement at the top of the stell sheet pile walls, the correct ones, as they were blocked like the struts on site. These horizontal displacements are located between the top and base of the stell sheet pile walls and increase with the depth of the excavation. For this excavation, it is the gravity unloading which induces the failure of the soil ground by means of an increase in the deviatoric stresses. A rigorous numerical modelling of such a structure is to prevent its failure mechanisms (depending on the stresses and displacements) and to be able to anticipate by taking the necessary solutions to avoid collapses during and after the construction. Observation of the horizontal displacement curves generated by stell sheet pile walls during excavation shows that a second bed (approximately to –5.75 m deep) of struts is necessary to prevent these displacements (phase 9). The transformation of the geometry and stiffness of the stell sheet pile walls to the retaining walls of an equivalent bending stiffness on the one hand and regular geometric shapes allowed in this paper to overcome the difficulties of modelling these stell sheet pile walls in 2D with irregular shapes. The results of this approach are satisfactory in view of the horizontal displacement curves obtained on the stell sheet pile walls compared by the measures.

**Fig 44:**
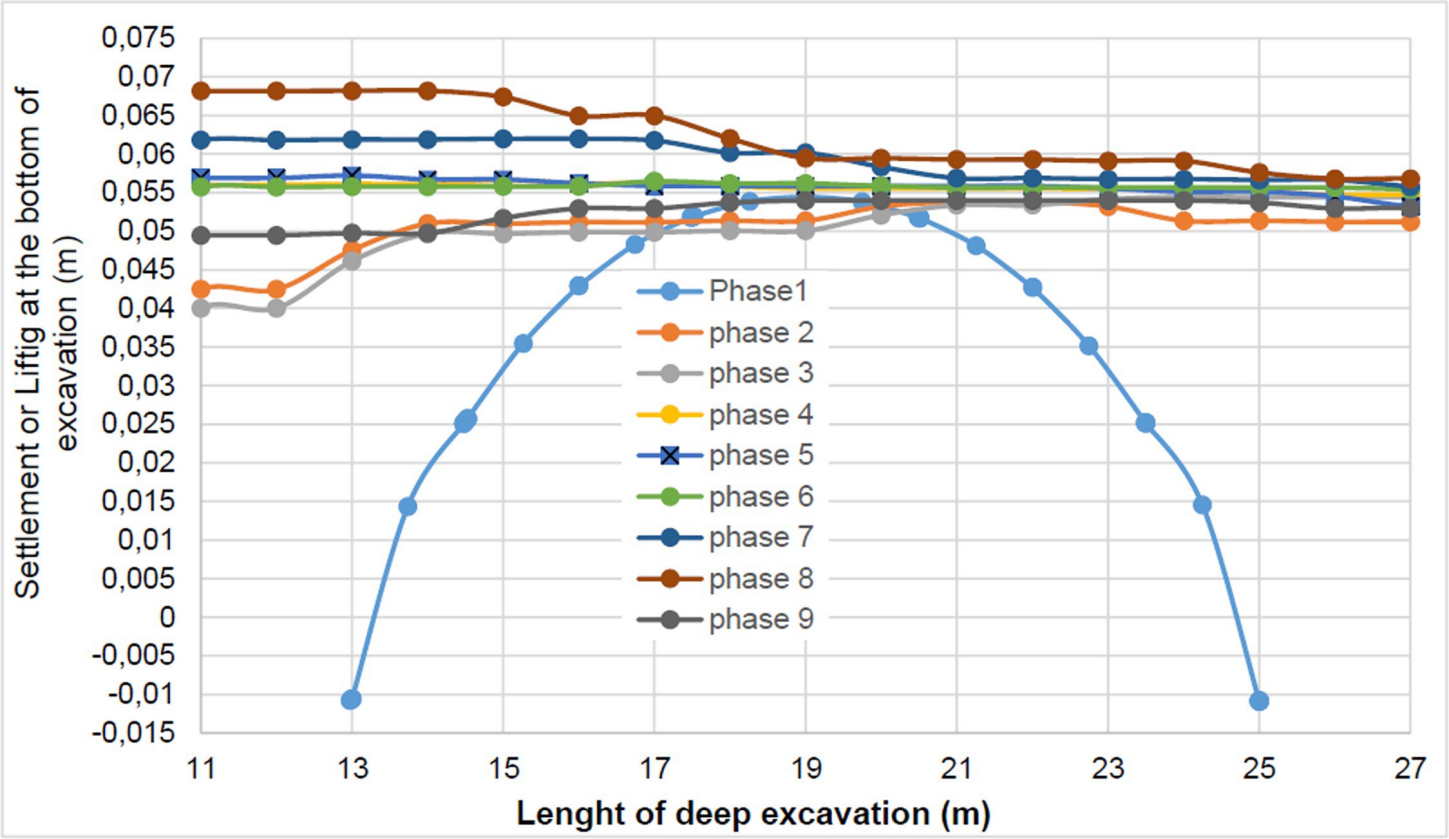
Lifting (m) at the bottom of excavation for phases 1 to 9.

**Fig 45:**
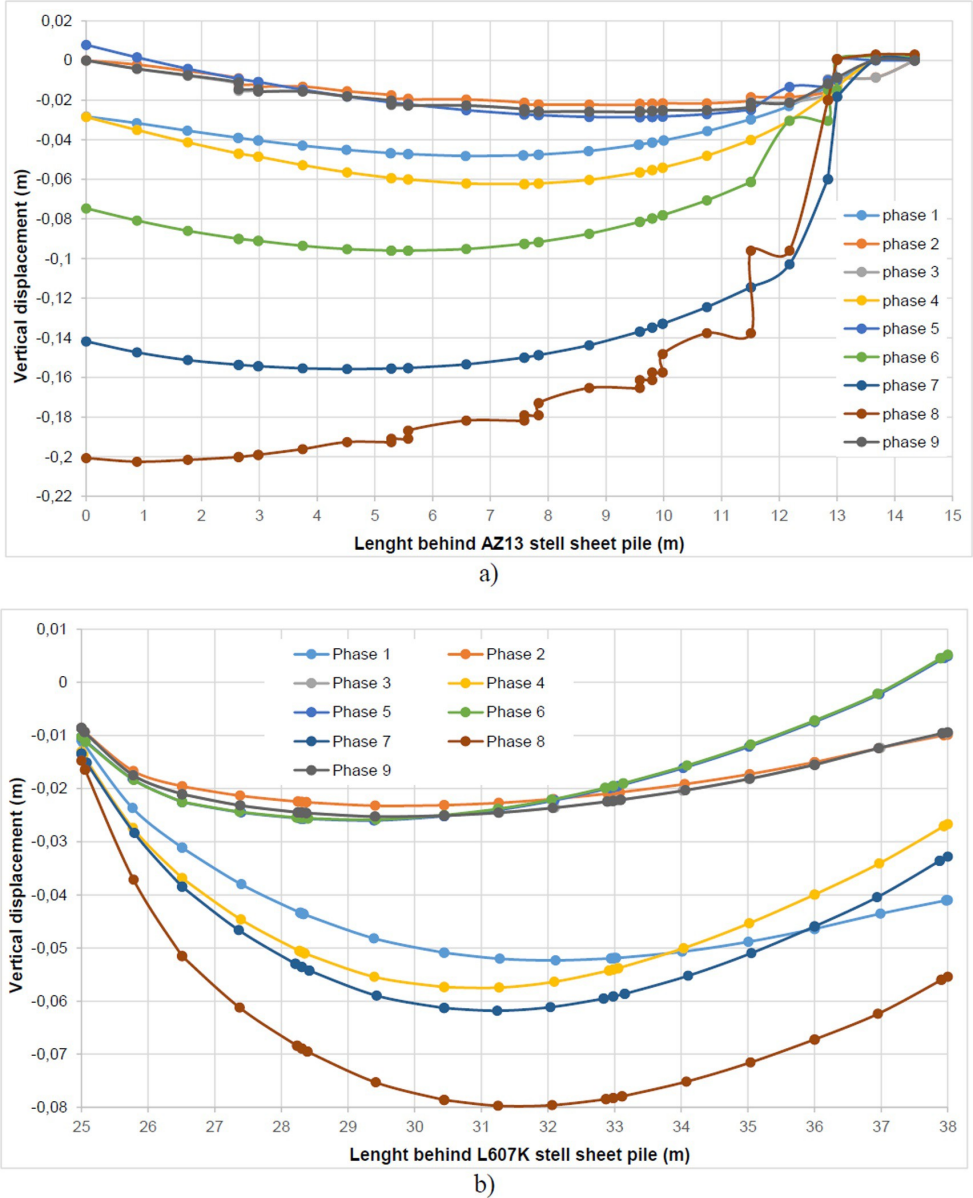
Settlement (m) behind the walls for phases 1 to 9: a) settlement behind AZ13 wall; b) settlement behind L607K wall.

The Drucker-Prager law used to describe the behavior of the soil models made it possible to realistically predict the response of the structure following the phases of excavation, lowering or fill the water level in the excavation. This paper shows that rigorous modelling (very fine mesh, behavior laws adapted to the materials, compliance with execution phases) realistically allows the response of the structure (Figs 44 and 45B). The results obtained in this study (Table 4 and Figs 5–45) are of the same order as those resulting from measurements and those obtained by various authors around the world (Table 5). Based on the state-of-the-art adapted to the retaining walls, the designer is able to make decisions that guarantee the durability of this structure according to the functional requirements and the expected lifetime. Such an approach saves large budgets for maintenance and repair operations. Rigorous numerical modelling makes it possible to obtain valid results at reasonable costs without waiting for long delays to draw lessons from full-scale experiments with structures that require very high time and budgets.

The stability calculations have been performed using Plaxis Finite Element code [84] in phase 1, concerning the dry excavation to– 4.0 m and phase 2; phase 3 and phase 8 concerning calculation of the long-term behavior of the structure with the gradual lowering of the water level to the bottom of the excavation (-7.0 m). In these stability calculations, the strength reduction method has been used in Plaxis software. The Factor of Safety is equal to 3.66 in phase 1; 4.15 in phase 2; 3.31 in phase 3 and 2.24 in phase 8 (see Figs 9B, 13B, 16B and 35B). In these adverse cases where the water level is—1.5m from the surface and at the bottom of the excavation, the shear failure safety factors of the excavation supports are greater than 1.5 guaranteeing the stability of the structure in its most critical phases.

## 6. Conclusion

This study improve the state-of-the-art requirements verification process for deep excavation. It is argued that the Finite Element method for excavation retained by stell sheet pile walls is a more powerful alternative to traditional limit equilibrium methods and its widespread use should now be standard in geotechnical practice. Cast3M is an FE code dedicated mainly to the calculation of stiff structures. At present, from a methodical, rigorous and sophisticated computation, we manage to solve complex geotechnical problems both respecting the functional requirements and the different stages of excavation/construction allowing to have the overall stability which guarantees the durability of the geotechnical structure in comfortable safety conditions. The results presented in this study were compared with those from measurements testifying to the reliability of our rigorous computation from the Cast3M code. Optimization numerical backcalculation results are proposed for retained walls design and construction on the basis of the horizontal displacements, earth and water pressures measurements. The transformation of the geometry and stiffness of the stell sheet pile walls to the retaining walls of an equivalent bending stiffness on the one hand and regular geometric shapes allowed in this paper to overcome the difficulties of modelling these stell sheet pile walls in 2D with irregular shapes. The results of this approach are satisfactory in view of the horizontal displacement curves obtained on the stell sheet pile walls compared by the measures. Numerical modelling makes it possible to carry out calculations that contribute to the optimization of geotechnical structures. Nevertheless, if they are poorly carried out, these calculations can lead to erroneous interpretations in the design of geotechnical structures and it is therefore more necessary than ever to know the most important aspects of numerical modelling. In this study, the horizontal deflection of the wall, the vertical displacement behind the walls, and the settlement of the excavation bottom are given. They have been compared by those obtained by various authors around the world and measurements.
